# Evaluation of double expression system for co-expression and co-immobilization of flavonoid glucosylation cascade

**DOI:** 10.1007/s00253-022-12259-5

**Published:** 2022-11-05

**Authors:** Agata Matera, Kinga Dulak, Sandra Sordon, Kacper Waśniewski, Ewa Huszcza, Jarosław Popłoński

**Affiliations:** Department of Food Chemistry and Biocatalysis, Wrocław University of Environmental and Life Sciences, C.K. Norwida 25, 50-375 Wrocław, Poland

**Keywords:** Co-expression, Co-immobilization, Biochanin A, Cascade reaction, Glucosyltransferase, Sucrose synthase, Biocatalysis, Flavonoids, Isoflavone

## Abstract

**Abstract:**

Glucosylation cascade consisting of Leloir glycosyltransferase and sucrose synthase with in situ regeneration system of expensive and low available nucleotide sugars is a game-changing strategy for enzyme-based production of glycoconjugates of relevant natural products. We designed a stepwise approach including co-expression and one-step purification and co-immobilization on glass-based EziG resins of sucrose synthase from *Glycine max* (*Gm*SuSy) with promiscuous glucosyltransferase YjiC from *Bacillus licheniformis* to produce efficient, robust, and versatile biocatalyst suited for preparative scale flavonoid glucosylation. The undertaken investigations identified optimal reaction conditions (30 °C, pH 7.5, and 10 mM Mg^2+^) and the best-suited carrier (EziG Opal). The prepared catalyst exhibited excellent reusability, retaining up to 96% of initial activity after 12 cycles of reactions. The semi-preparative glucosylation of poorly soluble isoflavone Biochanin A resulted in the production of 73 mg Sissotrin (Biochanin A 7-*O*-glucoside). Additionally, the evaluation of the designed double-controlled, monocistronic expression system with two independently induced promoters (*rhaBAD* and *trc*) brought beneficial information for dual-expression plasmid design.

**Key points:**

• *Simultaneous and titratable expression from two independent promoters is possible, although full control over the expression is limited*.

• *Designed catalyst managed to glucosylate poorly soluble isoflavone*.

• *The STY of Sissotrin using the designed catalyst reached 0.26 g/L∙h∙g of the resin*.

**Graphical Abstract:**

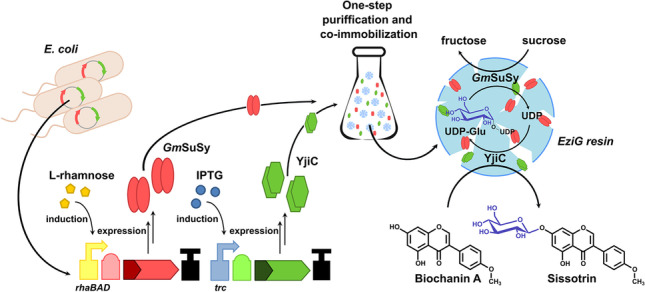

**Supplementary information:**

The online version contains supplementary material available at 10.1007/s00253-022-12259-5.

## Introduction

For the last few decades, biocatalysis has steadily gained prominence as an environmentally mild manner for the production of industrially relevant chemicals (Busacca et al. 2011; Sheldon and Brady 2022). Variable biological systems such as genetically engineered microorganisms, recombinant proteins, or new-to-nature enzymatic pathways are concurrently developed to deliver more and more efficient, robust, and easy-to-implement technologies. Enzymatic cascades in comparison to single-enzyme processes offer limited intermediate accumulation thus intermediate inhibition, fine volumetric productivity, and often independence from the addition of expensive and low-available cofactors (Santacoloma et al. 2011; France et al. 2017). The in vitro cascades and pathways are particularly important in the production and modification of agents with antimicrobial activities such as flavonoids, since they may disrupt the metabolism of living biocatalysts (Cushnie and Lamb 2011; Ahmad et al. 2012), and possibly decrease the outcome of the process.

Flavonoids, a plant secondary metabolite, due to proven antioxidant, anticancer, antibacterial, antiviral, cardioprotective, and flavour-regulatory properties are broadly used in the design of new pharmaceuticals and food additives (Panche et al. 2016; Koosha et al. 2016; Ullah et al. 2020). The polyphenolic structure lies behind prominent biological activities of flavonoids, but also contributes to their poor solubility and thus weak bioavailability (Heim et al. 2002; Chebil et al. 2007). However, flavonoid glycoconjugates, in comparison to their aglycones, have enhanced water solubility, stability, and altered pharmacokinetic parameters (Yang et al. 2018); therefore, glycosylation is an attractive and frequently undertaken route for functionalization of this class of compounds (Ji et al. 2020; Khodzhaieva et al. 2021). Nucleotide sugar-dependent Leloir glycosyltransferases (GT) are fine catalysts for highly regioselective and stereoselective glycosylation and are already broadly employed for the modification of natural products (Krasnova and Wong 2019; Mestrom et al. 2019). Due to the necessity of the addition of expensive and unstable nucleotide sugars (NDP-sugars) as sugar donors, the in situ recycling of NDP-sugars is essential for the cost-effectiveness of the application of a discussed class of enzymes (Sauerzapfe and Elling 2008; Mestrom et al. 2019). Among available pathways for the formation of NDP-sugars, sucrose synthase (SuSy) enables the reversible transfer of glucose from cheap sucrose to nucleotide (NDP), delivering the simplest and tailored solution for a continuous supply of required activated glucoside (Schmölzer et al. 2016). The SuSy-GT two-step glucosylation cascade has been already successively employed for the glucosylation of macrolides (Rupprath et al. 2007), diterpenes (Zhang et al. 2020), saponins (Ali et al. 2021; Chu et al. 2021), and flavonoids (Gutmann and Nidetzky 2016; Pei et al. 2020; Liu et al. 2021), both in soluble and heterogeneous (immobilized) form.

Enzyme immobilization on the surface of solid supports is an important technique in industrial applications of biocatalysis. It simplifies the usage of enzymes, improves their reaction efficiency, and enables the recycling of the catalyst after the process (Sheldon 2007; Califano and Costantini 2021). Especially co-immobilization, that is the immobilization of several enzymes on the same carrier particles, can enhance cascade reaction efficiency due to the spatial proximity effect on immobilized enzymes (Arana-Peña et al. 2021). The EziG carriers (EnginZyme AB, Stockholm, Sweden) developed for the immobilization of His_6x_-tagged enzymes are an attractive alternative to standard nitrilotriacetic acid (NTA) agarose resins, with advanced pore structure of glass-based particles, which enables the better mass transfer of reactants to solid material (Thompson et al. 2019).

Here we present a complex investigation of the co-expression and co-immobilization of the designed glucosylation cascade consisting of *Glycine max* sucrose synthase (*Gm*SuSy) and glucosyltransferase from *Bacillus licheniformis* (YjiC) and its application in the optimized semi-preparative scale glucosylation of poorly soluble isoflavone (Biochanin A). The research aimed at the development of a simplified strategy for the preparation of the reusable and robust catalyst tailored for efficient glucosylation of a variety of flavonoid compounds, due to the broad substrate promiscuity of employed GT (Pandey et al. 2014). Prepared by simultaneous purification and co-immobilization of both enzymes, the solid catalyst exhibited high immobilization yield and fine recovered activity. The co-immobilization process was supported by the co-expression of both enzymes in one *Escherichia coli* strain to decrease the enzyme production costs. Additionally, we attempted to create a monocistronic expression system with independent control of expression levels of desired enzymes, to avoid unfavorable disproportion of enzyme activities and thus maximize the cascade efficiency in the cell lysates and with immobilized catalyst. Moreover, our study brings new insights into the mechanisms of simultaneous co-expression from two distinctly induced promoters.

## Materials and methods

### Chemicals

Unless otherwise stated, all chemicals and medium compounds were bought from Sigma-Aldrich (St Louis, USA), Carbosynth (Compton, Berkshire, UK), or SERVA Electrophoresis Gmb (Heidelberg, Germany). Antibiotics were purchased from Cayman Chemical Company (Ellsworth, USA). Restriction enzymes (*Bsa*I-HFv2, *Bbs*I-HF), T4 DNA ligase, *Taq* PCR Kit, Plasmid Miniprep Kit, and all the rest necessary molecular biology reagents were bought from New England Biolabs Inc. (Ipswich, USA). Biochanin A was purchased from Carbosynth (Compton, Berkshire, UK). The UHPLC grade solvents used in this study were bought from Merck KGaA (Darmstadt, Germany).

### Plasmids construction

All genes, primers, plasmids, and strains used and constructed within this research are listed in Table S1. All strains harboring created plasmids were obtained by transformation of chemically competent *E. coli* DH5-alpha, *E. coli* 10-beta (for plasmid maintenance), or *E. coli* BL21 (DE3) (for protein production) cells. Positive clones were selected with blue-white screening using selective media supplemented with X-Gal (20 µg/mL) and appropriate antibiotic (ampicillin (100 mg/L), kanamycin (50 mg/L), or gentamicin (50 mg/L)), colony PCR procedure using *Taq* DNA polymerase and positive phenotype. The sequences of expression cassettes were confirmed by Macrogen Europe BV.

**Fig. 1:**
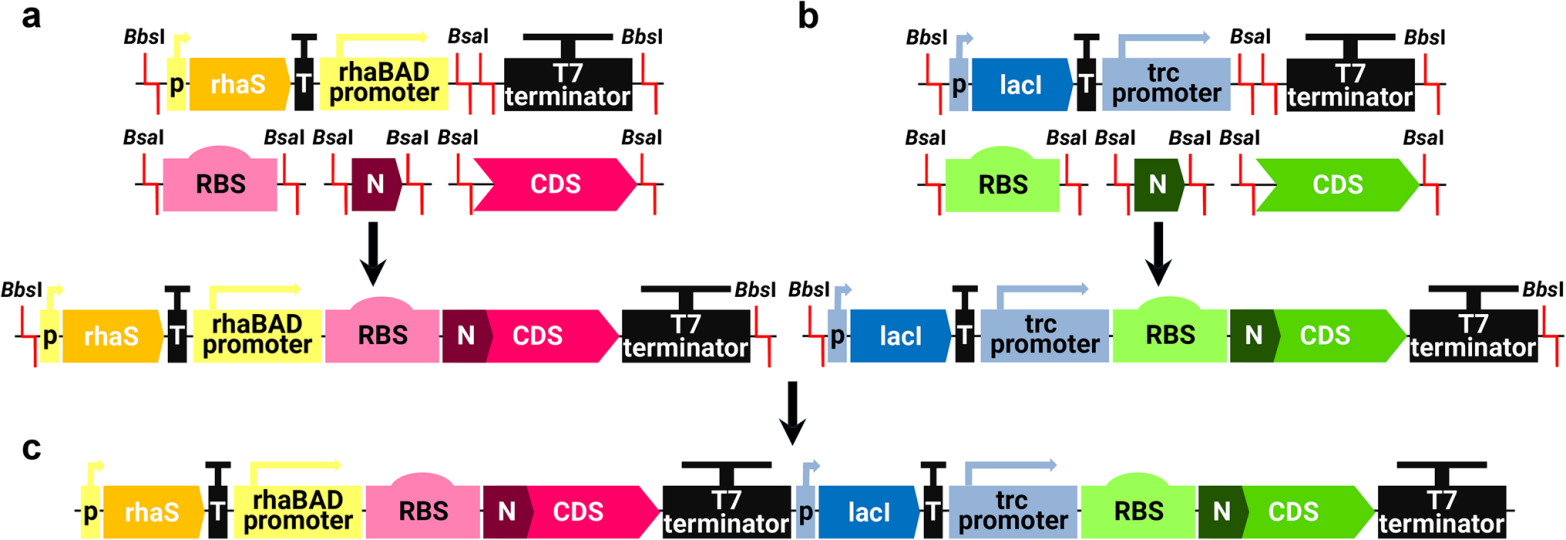
Schematic map of the construction of: **a** single gene RhaBAD expression cassette; **b** single gene Trc expression cassette; **c** RhaBADTrc double expression cassette. Red lines mark restriction enzymes cleavages. Abbreviation: CDS, coding sequence; p, promoter (pJ23103 for the *rhaS* expression; placQ for the *lacI* expression); T, terminator (T500 for the *rhaS* expression; T1 for the *lacI* expression); N, N-tag

Flanked by *Bbs*I restriction sites, RhaBAD and Trc empty expression cassettes (Fig. 1) including appropriate promoter, transcriptional regulator, and T7 terminator sequences were synthesized and cloned into pMA-T plasmids by Invitrogen GeneArt Gene Synthesis (Thermo Fisher Scientific, Waltham, MA, USA). Both plasmids were then digested by *Bbs*I restriction enzyme and cassettes were inserted into pSEVA23g19g1 and pSEVA23g19g2 vectors, respectively. Plasmids (pRhaBAD_12 and pTrc_23) served as backbone vectors in the following investigations.

Genes encoding *Gm*SuSy sucrose synthase and YjiC glucosyltransferase were codon-optimized for *E. coli*, synthesized with flanking *Bsa*I restriction sites, and cloned into the plasmids by Invitrogen GeneArt Gene Synthesis (Thermo Fisher Scientific, Waltham, MA, USA). Both genes were cut out by *Bsa*I-HFv2 restriction enzyme and inserted along with T7 RBS and N-His_6x_-tag to pRhaBAD_12 and pTrc_23 backbone vectors respectively. Plasmids (pRhaBAD-GmSuSy and pTrc-YjiC) were then digested by *Bbs*I-HF restriction enzyme and both inserted alongside into pSEVA63g19gA vector to create a monocistronic genetic configuration with independent induction systems for both genes (pRhaBAD-GmSuSy_Trc-YjiC).

Plasmid with *mCherry* coding sequence flanked with *Bsa*I restriction sites was purchased from Invitrogen GeneArt Gene Synthesis (Thermo Fisher Scientific, Waltham, MA, USA). Plasmid with *GFP* (green fluorescent protein) coding sequence flanked with *Bsa*I restriction sites was kindly provided by Juan Nogales’s research group (SBG, CSIC, Madrid). Both genes were proceeded in the same manner as *GmSuSy* and *yjiC* genes, which resulted in the construction of pRhaBAD-mCherry_Trc-GFP plasmid. Firstly, *mCherry* and *GFP* sequences were cut out by *Bsa*I-HFv2 restriction enzyme and inserted along with T7 RBS and N-His_6x_-tag sequences to pRhaBAD_12 and pTrc_23 backbone vectors, respectively. The expression cassettes were then digested by *Bbs*I-HF restriction enzyme and both inserted alongside into pSEVA63g19gA vector to create a monocistronic genetic configuration with independent induction systems for both genes (pRhaBAD-mCherry_Trc-GFP).

### Enzyme expression screening

*E. coli* BL21 (DE3) harboring pRhaBAD-GmSuSy_Trc-YjiC plasmid was grown overnight in a 100-mL Erlenmeyer flask containing 30 mL of lysogeny broth (LB) supplemented with 50 mg/L gentamicin in an incubator shaker (New Brunswick Innova 44, Eppendorf, Vienna, Austria) at 37 °C and 120 rpm agitation. The next day, five baffled 250 mL shaken flask with 60 mL of terrific broth (TB) containing 50 mg/L gentamicin were prepared. Each were inoculated with 10% *(v/v) *of overnight preculture and incubated at 37 °C and 120 rpm agitation until an optical density at 600 nm (OD_600_) reached around 0.6–0.8 value. Protein expression was then induced in each culture by the addition of different concentrations of L-rhamnose and IPTG (isopropyl β-D-1-thiogalactopyranoside) (Table 1).

**Table 1:** Tested concentrations of the inducers for the evaluation of the enzymes expression

Inducer	Concentration (mM)				
IPTG	0.01	0.1	1	1	1
L-rhamnose	25	25	25	12.5	1

Expression was carried out overnight in an incubator shaker at 25 °C and 120 rpm agitation. The cultures were then harvested by centrifugation (4000 g, 30 min, 4 °C) and the pellets were resuspended in 50 mM HEPES buffer (pH 7.5), digested by lysozyme (300 μg/mL) for 1.5 h at 4 °C, and disrupted by sonication on ice using Vibra-Cell Ultrasonic Liquid Processor VCX 130 (Sonics & Materials, Inc., Newtown, USA) for 2.5 min by the following procedure—5 s pulses and 5 s pauses at 80% amplitude. The cell lysates were then recovered by centrifugation (14000 g, 30 min, 4 °C) and their catalytic activity was evaluated using enzyme assays described below. Cell lysate from strain harboring pRhaBAD-GmSuSy_YjiC plasmid (Supplementary materials) and induced by 10 mM of L-rhamnose was used as a comparison of the expression from single and double controlled expression systems.

### In vivo fluorescence measurements

For the in vivo fluorescence measurements, *E. coli* BL21 (DE3) strain harboring pRhaBAD-mCherry_Trc-GFP plasmid was cultivated at 37 °C in the 96-well microplate in Synergy H1 microplate reader (BioTek Instruments, Vermont, USA) using 22.5 h continuous assay with 45 min intervals for shaking (15 s, 282 cpm), fluorescence (GFP: excitation 479 nm, emission 520 nm; mCherry: excitation 579 nm, emission 616 nm), and absorbance (OD_600_) measurements. The working volume in each well was 200 μL. Cultivation was carried out in a double concentrated M9 minimal medium (2xM9, Table S2) with enhanced buffering capacity (Azatian et al. 2020) due to pH dependence of GFP fluorescence (Doherty et al. 2010). The medium was supplemented with 50 mg/L gentamicin, 10*% (v/v)* of overnight preculture, 0.8% *(w/w)* glucose, and different concentrations of the inducers (Table 2).

**Table 2:** Tested concentrations of the inducers for *in vivo* fluorescence measurements

Inducer	Concentration (mM)										
IPTG	0	0.1	0.25	0.5	1	2.5	0.5	0.5	0.5	0.5	0.5
L-rhamnose	10	10	10	10	10	10	0	1	2.5	5	25

The uninduced culture was used as a control of the expression of fluorescent proteins. All variants were cultivated in three independent replicates. Fluorescence values were normalized against the OD_600_.

### Enzymes overexpression

*E. coli* BL21 (DE3) harboring pRhaBAD-GmSuSy_Trc-YjiC or pRhaBAD-GmSuSy_YjiC plasmid was grown overnight in a 100-mL Erlenmeyer flask containing 30 mL of LB supplemented with 50 mg/L gentamicin in an incubator shaker (New Brunswick Innova 44, Eppendorf, Vienna, Austria) at 37 °C and 120 rpm agitation. The next day, 500 mL of TB containing 50 mg/L gentamicin was inoculated with 10% *(v/v)* of overnight preculture and incubated in a baffled 2 L shaken flask at 37 °C and 120 rpm agitation until an OD_600_ reached around 0.6–0.8 value. Protein expression was then induced by the addition of 10 mM of L-rhamnose and 1 mM of IPTG (pRhaBAD-GmSuSy_Trc-YjiC) or 25 mM of L-rhamnose (pRhaBAD-GmSuSy_YjiC). Expression and sonication was performed as described above in the enzyme expression screening section. The cell lysate was then recovered by centrifugation (14000 g, 30 min, 4 °C) and stored at 4 °C.

### Enzymes co-immobilization

Co-immobilization of the enzymes was performed on standard agarose based resin coated with nickel ions (Ni-agarose resin) and EziG Amber, Coral, and Opal resins. Each resin was prepared following the manufacturer protocol (see Supplementary materials) using HEPES buffer (50 mM, 50 mM KCl, 300 mM NaCl, pH 7.5). Next, *E. coli* cell lysate containing both enzymes and 20 mM of imidazole was added to the prepared resins. Immobilizations were carried out on ice on the orbital shaker (ELMI, Riga, Latvia) at 100 rpm agitation for 2 h. Then suspensions were centrifuged at 500 g for 5 min, supernatants were removed, and resins were washed two times with wash buffer (50 mM HEPES, 50 mM KCl, 300 mM NaCl, 5 mM imidazole, pH 7.5). Supernatants and washing solutions were collected for protein and activity measurements. The concentration of the proteins was evaluated using Bicinchoninic Acid Kit for protein determination (Sigma-Aldrich, St Louis, USA) and bovine serum albumin as a reference for the calibration curve. The enzymatic activity was determined by the standard cascade assay (described below). Solutions were also analyzed by the SDS-PAGE protocol (Laemmli 1970) using Color Prestained Protein Standard Broad Range (10–250 kDa) (New England Biolabs Inc. Ipswich, USA) as a molecular weight marker. Prepared resins were resuspended in HEPES buffer (50 mM, 50 mM KCl, pH 7.5) and stored at 4 °C.

The protein capacity of each resin was calculated as the difference between the protein amount in cell lysate before immobilization and cell lysate and wash solutions after immobilization. The enzyme activity bound to the resin (bound activity) was calculated as a difference between the cell lysate activity before (A_0_) and after the immobilization process (A), divided by the mass of the employed resin (g). The bound activity is expressed as U/g. Immobilization yield (%) was calculated as a ratio between ΔA (= A_0_-A) and A_0_. The observable activity of the solid catalyst was measured directly by the standard cascade assay and expressed as U/g. Recovered activity (%) is the ratio between the observable and the bound activity of the solid catalyst.

### Enzyme assays

All reactions were performed in 0.8 mL total volume in 2-mL tubes at 500 rpm (soluble enzymes) or 1000 rpm (immobilized enzymes) agitation in a shaker (Eppendorf ThermoMixer C) for 15 min. The reactions were terminated by heating at 100 °C for 5 min and centrifuged (21300 g, 5 min) to remove the solid materials (precipitated proteins, or resin). The supernatants were analyzed via the described below protocols. The reaction mixtures lacking the catalyst served as a control.

#### YjiC

The general glucosyltransferase reaction mixture contained 0.5 mM uridine diphosphate glucose (UDP-glucose), 0.1 mM *p*-nitrophenol (*p*NP), 50 mM KCl, and 40 μL of the cell lysate or 40 μL of the suspension of co-immobilized enzymes (which corresponded to 2.8 mg of the dry resin). The reaction progress was analyzed by the measurements of the *p*NP absorbance at 405 nm on the Synergy H1 microplate reader (BioTek Instruments, Vermont, USA) in 200 μL working volume. The concentration of the *p*NP was calculated in reference to the standard curve. One unit (U) of the catalyst activity is defined as the enzyme amount releasing 1 μmol *p*NP β-D-glucoside/min under specific conditions.

#### *Gm*SuSy

The general sucrose synthase reaction mixture contained 2 mM uridine diphosphate (UDP), 500 mM sucrose, 50 mM KCl, and 40 μL of the cell lysate or 40 μL of the suspension of co-immobilized enzymes (which corresponded to 2.8 mg of the dry resin). The reaction progress was analyzed by the detection of the fructose by the modified bicinchoninic acid (BCA) method (Waffenschmidt and Jaenicke 1987; Cerdobbel et al. 2010), which was evaluated for sucrose interference (Table S3). Reaction samples (10 μL) were added to the 190 μL of the assay solution (see Supplementary materials) and heated at 100 °C for 5 min. After cooling to room temperature, the absorbance at 560 nm was measured on the Synergy H1 microplate reader (BioTek Instruments, Vermont, USA). One unit (U) of the catalyst activity is defined as the enzyme amount releasing 1 μmol fructose/min under specific conditions.

#### *Cascade*

The general cascade reaction mixture contained 0.5 mM UDP, 500 mM sucrose, 0.1 mM *p*NP, 50 mM KCl, and 40 μL of the cell lysate or 40 μL of the suspension of co-immobilized enzymes (which corresponded to 2.8 mg of the dry resin). The reaction progress was analyzed in the same manner as glucosyltransferase reactions. One unit (U) of the catalyst activity is defined as the enzyme amount releasing 1 μmol *p*NP β-D-glucoside/min under specific conditions.

### Enzyme characterization

The effect of the pH on enzyme activity was performed at 30 °C in the pH range 5.5–9.5 using the following buffers: 50 mM sodium citrate (pH 5.5, 6.5), 50 mM HEPES (pH 7.5, 8.5), and 50 mM glycine–NaOH (pH 9.5). The dependence of the enzyme activity on the temperature was checked at 20, 30, 40, and 50 °C at 50 mM HEPES buffer (pH 7.5). The enzyme activity at various Mg^2+^ ions concentrations (0.1, 1, 5, 10, and 50 mM) was evaluated at 40 °C and 50 mM HEPES buffer (pH 7.5). Enzyme stability was assessed at different pH values (6.5, 7, 7.5, 8, 8.5) and different temperatures (30, 35, 40, 45, and 50 °C) by incubation for 6 and 24 h in 50 mM HEPES buffer (50 mM KCl, 10 mM MgCl_2_, 500 mM sucrose). The residual cascade activity was checked using a standard cascade assay at 40 °C and was calculated as the percentage of the activity at the beginning of the incubation. In the all above-described characterizations, cell lysates and enzymes co-immobilized on Ni-agarose resin were used. Each test was performed in triplicates.

### Reusability of the co-immobilized cascade

Reactions were performed in 50 mM HEPES buffer (pH 7.5) containing 30 mg of solid catalyst, 0.5 mM UDP, 500 mM sucrose, 0.1 mM *p*NP, 50 mM KCl, and 10 mM MgCl_2_ in 0.6 mL total volume in sealed spin columns (Sigma-Aldrich, St Louis, USA) inserted into 2-mL tubes at 1000 rpm agitation in a shaker (Eppendorf ThermoMixer C) at 40 °C for 10 min. At the end of each cycle of reaction, columns were open, reaction mixtures were centrifuged (1000 g, 2 min) and fresh reaction mixtures were loaded to resealed spin columns for the consecutive cycle of catalysis. Filtrates were analyzed in the same manner as in the glucosyltransferase assay. Each carrier was reused 12 times in triplicates. The residual cascade activity was calculated as the percentage of the activity at the first cycle of reaction, separately for each resin.

### Fed-batch conversion of Biochanin A

The semi-preparative reaction was performed using co-immobilized on EziG Opal resin enzymes, in a total volume of 40 mL in 50-mL Eppendorf falcon in an incubator shaker (New Brunswick Innova 44, Eppendorf, Vienna, Austria, 30 °C, 120 rpm) for 48 h. Reaction was carried out at 50 mM HEPES buffer (pH 7.5) containing 0.5 mM UDP, 500 mM sucrose, 50 mM KCl, 10 mM MgCl_2_, 10% *(v/v)* DMSO (dimethyl sulfoxide), 150 mg of solid carrier, and 68.75 mg Biochanin A in total. Biochanin A dissolved in DMSO (64.5 mM stock) was stepwise added to the reaction at 0, 2, 4, 6, 8, and 24 h. Reaction samples (20 μL) were withdrawn hourly for the first 8 h and then after 24, 25, 26, and 48 h of the catalysis, extracted with 100 μL of ethyl acetate, and centrifuged (21300 g, 5 min). Thirty microliters of organic fractions was diluted in 170 μL of methanol and analyzed using UHPLC performed on Dionex Ultimate 3000 UHPLC + instrument (Thermo Fisher Scientific, Waltham, MA, USA) equipped with a DGP-3600A dual pump liquid control module, a TCC-3200 thermostated column compartment, a WPS-3000 autosampler, a diode array detector (DAD), and an analytical C-18 Acclaim RSLC Polar Advantage II (2.2 μm, 2.1 × 100 mm, Thermo Fisher Scientific) column, thermostated at 40 °C with 0.1% formic acid solution in water (A) and 0.1% formic acid solution in acetonitrile (B) as mobile phase in gradient elution program (0–3 min: 15–98% B; 3–4.2 min: 98% B; 4.2–4.4 min: 98–15% B; 4.4–6 min: 15% B) at 0.7 mL/min flow. The system control and data acquisition were done using Chromeleon 6.80 software (Dionex, Sunnyvale, USA). The detection was carried out at 280 nm, and the identification of the substrate and product was based on the retention time and UV spectrum of authentic standard compounds and product structure was also confirmed by NMR spectra analysis (Supplementary materials). The total turnover number (TTN) was calculated as the ratio of moles of generated product (Sissotrin) divided by the moles of UDP added to the reaction.

### Statistical analysis

The means, standard deviations of the mean, and Pearson’s correlation coefficients (PCC) were calculated from triplicate experiments using Statistica software (version 13.3).

### Accession numbers

Synthetic genes used in the study were deposited in NCBI Genbank database under accession numbers: OP381218 for Sucrose synthase and OP381219 for YjiC glycosyltransferase.

## Results

Our approach to create an efficient and reusable biocatalyst for glucosylation of flavonoids, which also requires minimum steps of preparation, was to optimize the reaction conditions, select the most robust carrier, and design an expression system for easy and convenient biocatalyst preparation.

### Plasmid construction and validation of double expression system

The backbone of the designed plasmids was based on the SEVA (Standard European Vector Architecture) (Martínez-García et al. 2020) which is compatible with the Golden Standard modular cloning (GS MoClo) assembly (Blázquez et al. 2022). Our experiments with vector design and construction started from a single expression system, with two genes (GT, SuSy) under the control of the same *rhaBAD* promoter; however, initial activity tests displayed a high difference in activity between two enzymes (~0.05 U of YjiC and ~2.0 U of *Gm*SuSy). Hence, we decided to create a vector that could independently control the expression of two genes to alleviate the activity gap. At the first stage of the formation of a functional double controlled expression system, consisting of two independently induced transcriptional units, plasmids with a single gene expression cassette were constructed—pRhaBAD_12 and pTrc_23 (Fig. 1).

pRhaBAD_12 vector contains tightly regulated *E. coli* rhamnose-inducible *rhaBAD* promoter, regulated by RhaS transcriptional factor (TF), which in the presence of L-rhamnose induces expression from the *rhaBAD* promoter (Fig. 1a). Within this investigation, a decoupled *rhaBAD* regulatory system was used, requiring only one (RhaS) of two existing transcriptional factors (RhaR and RhaS), since it was proved that such simplified expression mechanism has the same characteristics as a native system, and exhibits a broader range of acceptable inducers (Kelly et al. 2016). pTrc_23 vector contains a strong, hybrid *trc* promoter under the control of LacI TF, which represses expression from the *trc* promoter in the absence of IPTG (or other allolactose analogues) (Brosius et al. 1985). To co-express *Gm*SuSy sucrose synthase with YjiC glucosyltransferase, their coding sequences were cloned into pRhaBAD_12 and pTrc_23 vectors, respectively. Next, thanks to compatible fusion sites, both transcriptional units were incorporated into a single vector creating the final expression plasmid (pRhaBAD-GmSuSy_Trc-YjiC) that enabled the expression of the *Gm*Susy and YjiC induced by L-rhamnose and IPTG, respectively.

**Fig. 2:**
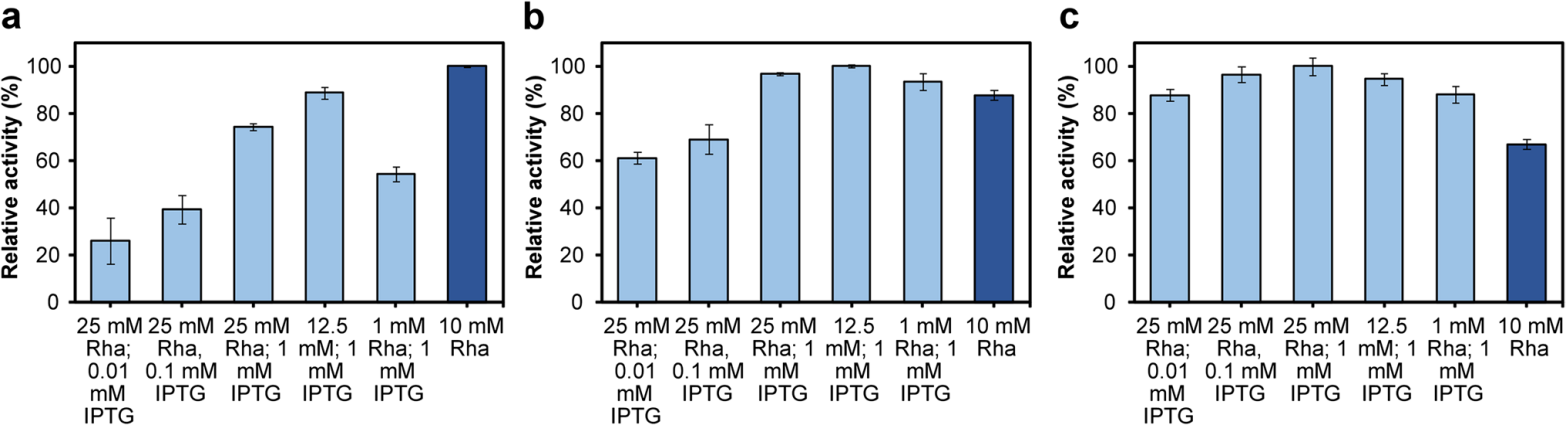
The relative activity of **a** cascade, **b** YjiC, and **c** *Gm*SuSy in the cell lysates obtained from differently induced cultures of strain harboring pRhaBAD-GmSuSy_Trc-YjiC plasmid (light blue bars) and pRhaBAD-GmSuSy_YjiC plasmid (dark blue bars) as a comparison of the expression from single and double controlled expression systems. The highest activities at each test were set as 100%. Error bars represent the standard deviations obtained from three individual replicates Abbreviation: Rha, L-rhamnose

To choose optimal inducers concentrations, which would guarantee the best enzyme ratio, a screening test of the enzyme activities in cell lysates obtained from differently induced cultures (see Table 1) was performed. The activities of the YjiC, *Gm*SuSy, and cascade reaction were assessed separately. For GT and cascade analysis, a glucosylation of the *p*NP was employed (Fig. S1, a). *p*NP displays an absorption maximum at 405 nm which disappears with the formation of the colorless *p*NP β-D-glucoside (Burchell and Weatherill 1981). This enables the evaluation of reaction progress through the absorbance measurements. Sucrose synthase activity was assessed analyzing fructose concentration, which is released in the transglycosylation of sucrose with UDP (Fig. S1, b). The results indicated the correlation between the enzyme’s activity and different inducer’s concentration. The cell lysates from cultures induced with higher concentrations of IPTG displayed not only better cascade and GT activity (due to stronger YjiC overexpression) but also slightly better SuSy effectiveness (Fig. 2), despite the fact of constant L-rhamnose concentration (Fig. 2).

Moreover, the best cascade and YjiC relative activity were achieved by the cell lysate induced with moderate (12.5 mM), not the highest tested (25 mM) L-rhamnose concentration (Fig. 2). Furthermore, comparing results from double and single expression systems, the highest cascade effectiveness was observed in the cell lysate from a strain where both genes were expressed from *rhaBAD* promoters. At the same time, YjiC and *Gm*SuSy activities in the aforementioned cell lysate were lower than in other tested protein extracts, what was unexpected and clearly indicated that more is not always better (Fig. 2). The single controlled expression vector was further applied to immobilization and reaction optimization experiments, although we decided to explore a bit more the double controlled expression vector.

**Fig. 3:**
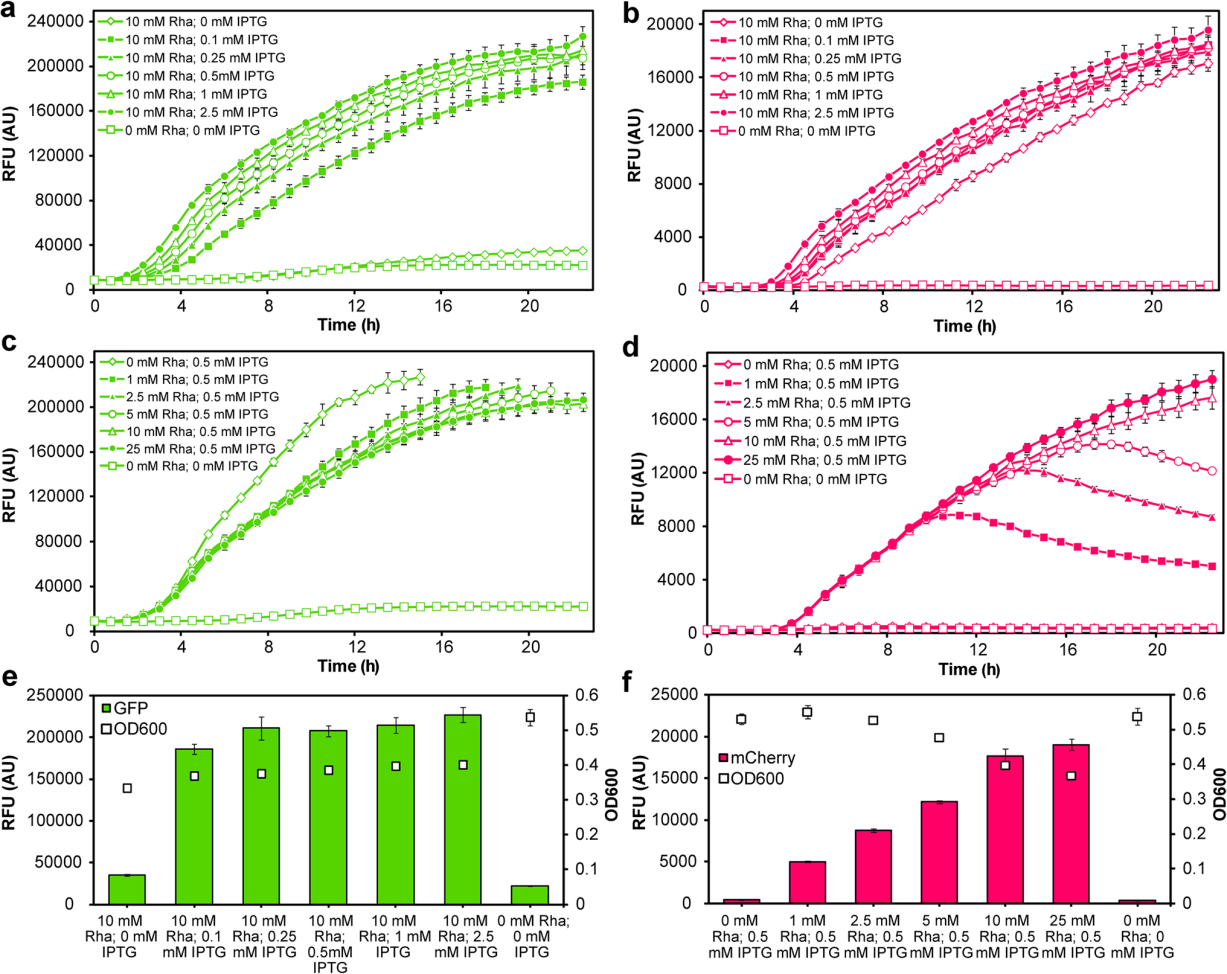
Expression of the fluorescent proteins GFP (**a**, **c, e**) and mCherry (**b**, **d, f**) in the liquid cultures of *E. coli* strain harboring pRhaBAD-mCherry_Trc-GFP plasmid, supplemented with different concentrations of the inducers. Charts **a**, **b, e** and **c**, **d, f** display measurements of the same cultures (see Materials and methods section). Measurements of the GFP fluorescence in the cultures supplemented with different amounts of L-rhamnose (**c**) were stopped when the intensity of the fluorescence exceeded the measurement capabilities of the equipment. **e** The final GFP fluorescence and OD_600_ after 22.5 h of cultivation. **f** The final mCherry fluorescence and OD_600_ after 22.5 h of cultivation. Error bars represent the standard deviations obtained from three individual replicates. Fluorescence values were normalized against the OD_600_ of the cultures

To understand the unusual co-action of IPTG in our double-controlled expression vector, we decided to validate the designed double expression system. Two fluorescent proteins (mCherry and GFP) with no overlapping excitation and emission spectra (Cormack et al. 1996; Shaner et al. 2004) were employed as model proteins not limited by activity or kinetic constraints and easily detectable. Their coding sequences were processed as the *GmSuSy* and *YjiC* genes, which resulted in the construction of pRhaBAD-mCherry_Trc-GFP expression plasmid—with overexpression of mCherry induced by L-rhamnose and GFP by IPTG addition. The dynamic range of simultaneous expression from both transcriptional units was assessed by the fluorescence measurements of liquid cultures supplemented with different amounts of the inducers (see Table 2). Cultures of *E. coli* strain harboring pRhaBAD-mCherry_Trc-GFP plasmid were induced in two sets—first with fixed L-rhamnose amount (10 mM) and increasing IPTG concentration (0–2.5 mM) and second with constant IPTG amount (0.5 mM) and increasing L-rhamnose concentration (0–25 mM). Measurements revealed a strong and stable expression of the GFP from *trc* promoter, regardless of the added IPTG amount, but with a positive correlation between fluorescence and inducer concentration (Fig. 3a, e, PCC for the measurements at 22.5 h equals 0.5056, *p *= 0.032). Some basal expression in the uninduced samples was also observed (Fig. 3a, e).

Interestingly, in the samples with stronger IPTG induction, a higher level of mCherry fluorescence was detected, despite the fact of the identical L-rhamnose concentration in relevant samples (Fig. 3b, PCC between mCherry fluorescence at 22.5 h and IPTG concentration equals 0.5503, *p* = 0.018). On the other hand, mCherry expression in cultures supplemented with various L-rhamnose concentrations presented a good scope of the induction range (PCC between mCherry fluorescence at 22.5 h and L-rhamnose concentration equals 0.8289, *p* < 0.000) and marginal basal expression (Fig. 3d, f). Results indicate also that the saturation of the *rhaBAD* promoter activity was reached around 10–25 mM of L-rhamnose concentration (Fig. 3d). Furthermore, in samples with lower L-rhamnose concentration, higher GFP fluorescence was observed (PCC between fluorescence at 15 h and L-rhamnose concentration equals −0.5664, *p* = 0.014), despite equal IPTG amount in relevant samples (Fig. 3c).

### Characterization of the biocatalysts

**Fig. 4:**
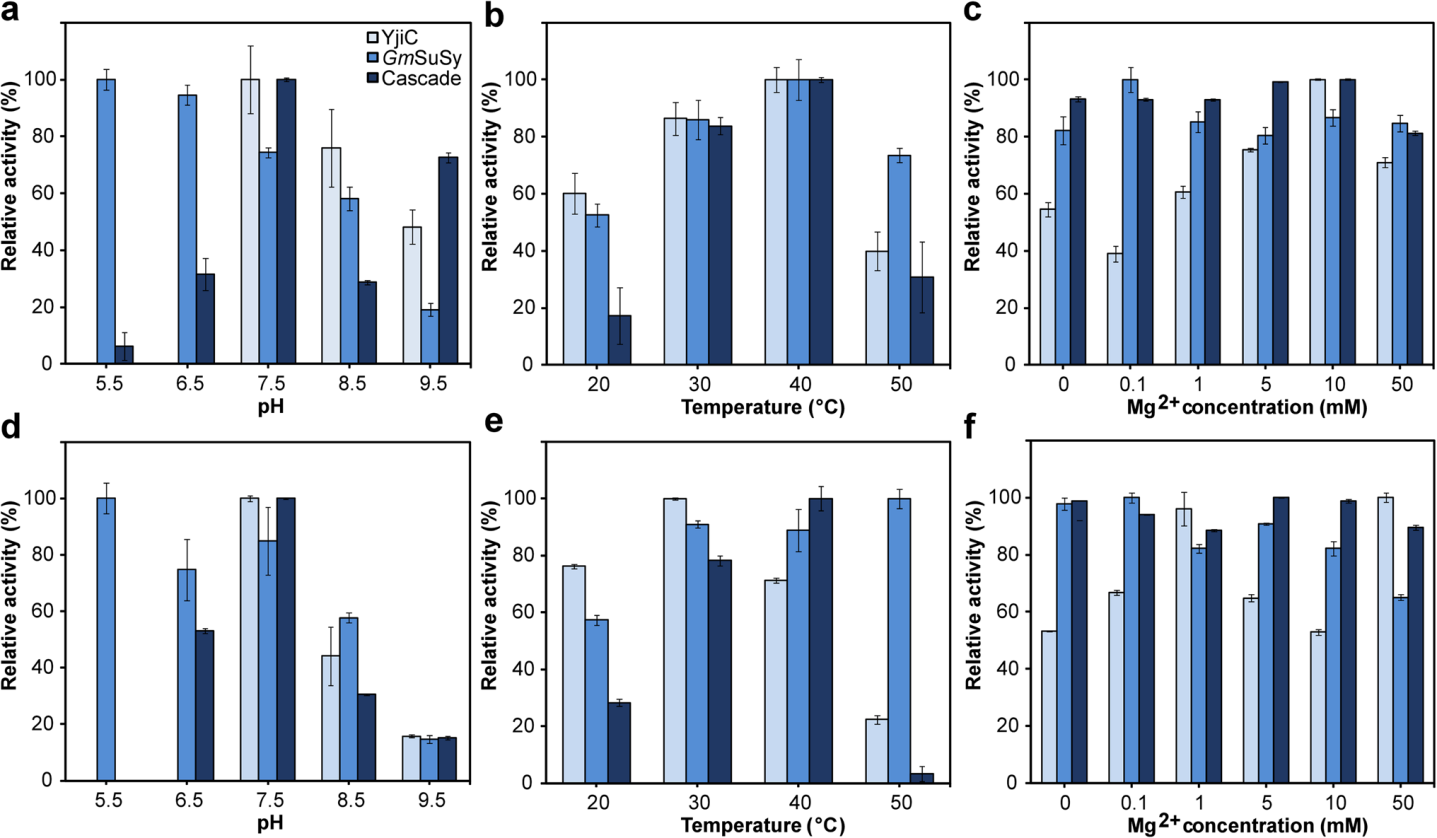
Effect of the pH (**a**, **d**), temperature (**b**, **e**), and Mg^2+^ concentration (**c**, **f**) on the activity of the soluble (**a**, **b**, **c**) and co-immobilized enzymes (**d**, **e**, **f**). Reactions were performed separately for YjiC (light blue bars), *Gm*SuSy (blue bars), and the cascade (dark blue bars). Used buffers: 50 mM sodium citrate (pH 5.5, 6.5), 50 mM HEPES (pH 7.5, 8.5), and 50 mM glycine–NaOH (pH 9.5). The highest activities at each test and each biocatalyst were set as 100%. Error bars represent the standard deviations obtained from three individual replicates

To select the optimal reaction conditions for larger-scale glucosylation and to compare the activity of soluble and co-immobilized enzymes, several tests were performed. The effect of the pH, temperature, and Mg^2+^ ion concentration on the activity of the soluble and co-immobilized enzymes was assessed separately for YjiC glucosyltransferase, *Gm*SuSy sucrose synthase, and coupled reaction at 30 °C. Soluble and immobilized YjiC displayed the highest activity at pH 7.5 (Fig. 4a, d). Sucrose synthase was the most active at 5.5 pH and gradually lost activity along with increased pH value (Fig. 4a, d). The cascade demonstrated a wider pH range of glucosylation in comparison to a single enzyme reaction (Fig. 4a, d). Surprisingly, greater shifts in the activity between each pH were observed for immobilized than soluble enzymes (Fig. 4a, d).

Soluble YjiC, *Gm*SuSy, and the cascade exhibited the highest activity at 40 °C (Fig. 4b). What is worth to note, the immobilization shifted the GT’s temperature optimum to 30 °C and SuSy’s to 50 °C but does not prevent loss of the activity of the glucosyltransferase above 40 °C (Fig. 4b, e). Both sucrose synthase and the cascade activity was not relevantly affected by the various Mg^2+^ concentration (Fig. 4c). The most sensitive for the changes in the magnesium ions concentration was YjiC glucosyltransferase (Fig. 4c, f).

**Fig. 5:**
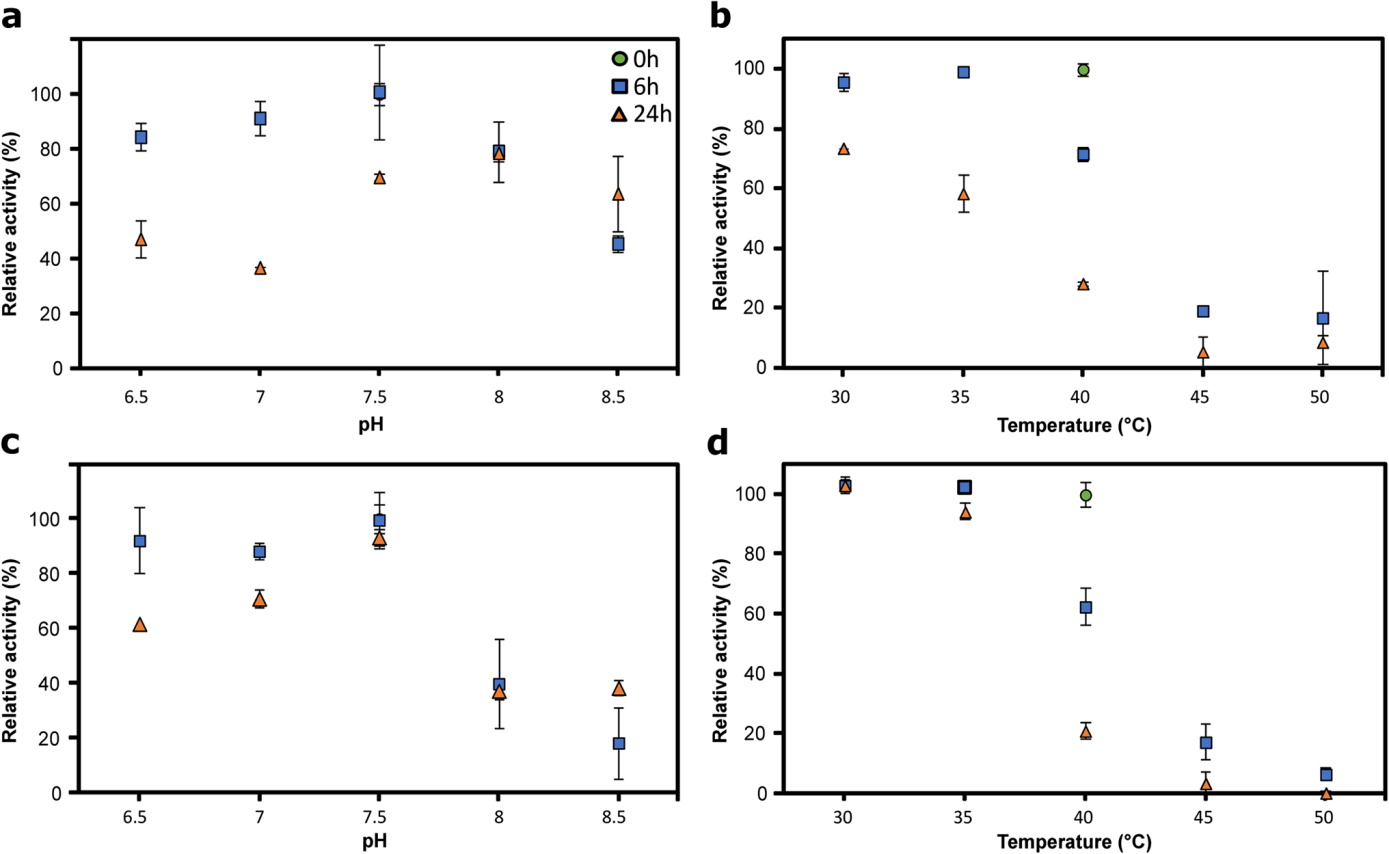
The stability at different pH (**a**, **c**) and different temperatures (**b**, **d**) of soluble (**a**, **b**) and co-immobilized (**c**, **d**) enzymes. The remaining activities were measured after 6 (blue squares) and 24 h (orange triangles) of incubation in tested conditions. The activity at 0 h (green circles) was set as a 100%. Error bars represent the standard deviations obtained from three individual replicates

The identification of the optimal cascade reaction conditions was followed by the assessment of the enzyme stability in the different temperatures and pH values to select the reaction settings guaranteeing not only the best operational activity but also fine durability of the enzymes, which is especially important for larger scale reactions and the reusability of the immobilized catalyst (Fig. 5).

The residual cascade activity assessments indicated that soluble enzymes displayed slightly better stability in a more alkaline environment (pH 8 and 8.5), while the co-immobilized catalyst was more stable in a more acidic pH values (pH 6.5–7.5) (Fig. 5a, c). Furthermore, the co-immobilization visibly improved cascade stability at 30 and 35 °C (at 30 °C no activity loss was detected) but did not prevent loss of activity above 40 °C (Fig. 5b, d). For further experiments, 30 °C, pH 7.5, and 10 mM Mg^2+^ were selected as the best cascade reaction conditions.

### Enzyme co-immobilization

The co-immobilization of the His_6x_-tagged enzymes was performed on four resins—agarose-based resin coated with Ni^2+^ and EziG carriers with controlled porosity glass (CPG) as a core and various surface properties: semi-hydrophilic Amber, hydrophobic Coral, and hydrophilic Opal resin, each containing chelated Fe^3+^ ions (kindly provided by the company as test samples). Enzymes were co-immobilized directly from the cell lysate prepared from *E. coli* strain harboring pRhaBAD-GmSuSy_YjiC plasmid. The highest protein capacity was reached by the EziG Amber and Coral resins, yet the best observable activity of the immobilized catalyst was attained for EziG Opal resin, suggesting higher selectivity of this carrier (Table 3). The agarose-based resin exhibited the lowest protein capacity, immobilization yield, observable activity, and space–time yield (STY) (Table 3). All carriers showed comparable recovered activity (Table 3). Calculated space–time yields for *p*NP β-D-glucoside of each resin indicated the best catalytic properties of the cascade immobilized on Opal resin (Table 3).

**Table 3:** Parameters of the immobilization process and the cascade co-immobilized on different resins. Given STY was calculated for the 1st cycle of the recyclability test

Resin type	Protein adsorption (mg/g dry resin)	Bound activity (U/g)	Immobilization yield (%)	Observable activity (U/g)	Recovered activity (%)	STY (g/L∙h∙g of resin)
Ni-agarose	2.6	0.29	48	0.15	52	2.71
EziG Amber	28.7	0.58	98	0.24	42	4.41
EziG Coral	26.7	0.52	88	0.27	52	4.93
EziG Opal	17.4	0.59	100	0.32	53	5.70

The SDS-PAGE analysis of the cell lysates and wash solutions, performed after the immobilization procedure, revealed high uptake of the His_6x_-tagged proteins by the EziG carriers, in comparison to agarose-based resin (Fig. S2). Interestingly, YjiC was immobilized almost completely by the EziG carriers, while a band corresponding to *Gm*SuSy is visible in all cell lysates after immobilization (Fig. S2).

**Fig. 6:**
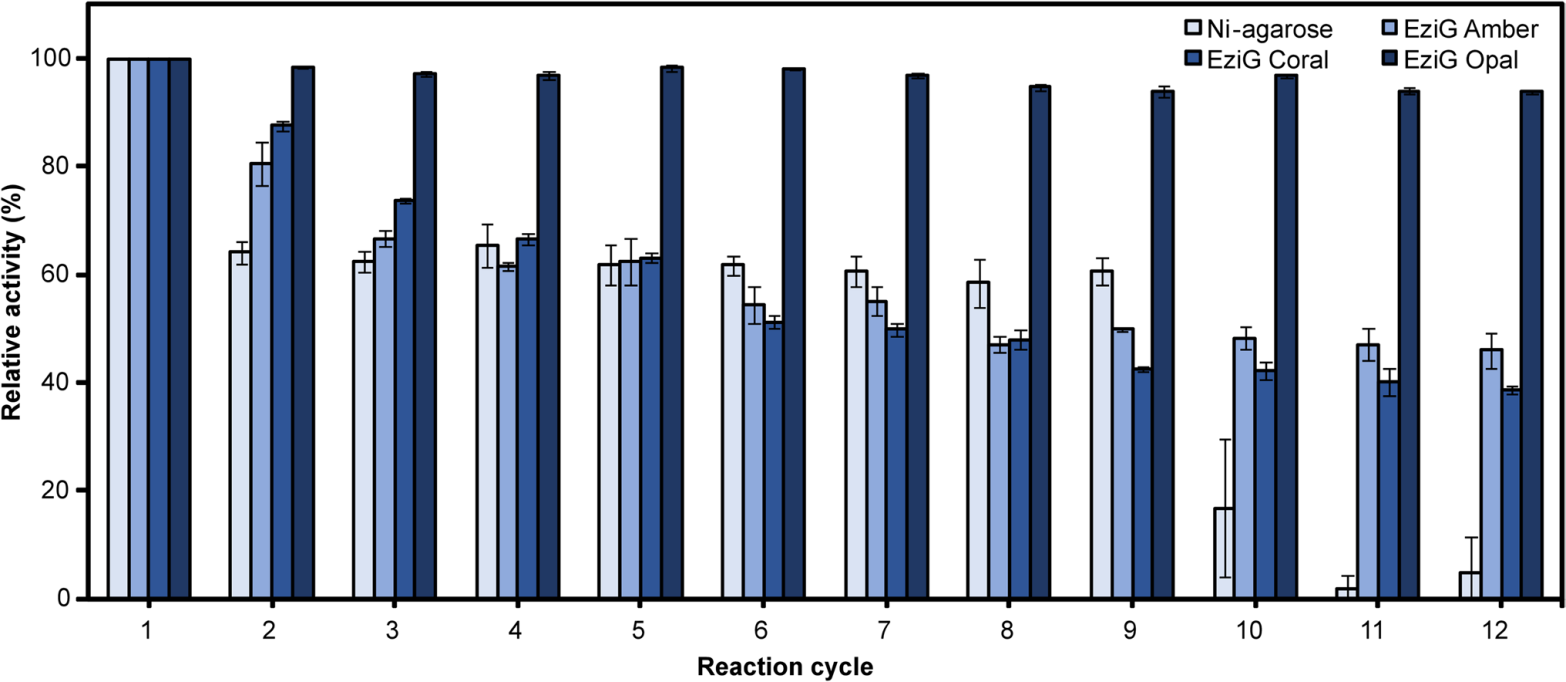
The reusability of the cascade immobilized on different resins. Error bars represent the standard deviations obtained from three individual replicates. The activity at 1st cycle was set as a 100%

The reusability of each solid catalyst was evaluated by performing several cycles of the standard cascade reaction assay. The best operational stability after 12 cycles of reaction displayed the cascade immobilized on Opal resin, losing only 6% of the initial activity (Fig. 6). Amber and Coral resins exhibited moderate reusability properties, losing 54% and 61% of the initial activity, respectively (Fig. 6). The activity of the cascade immobilized on Ni-agarose resin felt drastically after the 9th reaction cycle, and only 5% of initial activity was detected at the end of the experiment (Fig. 6).

### Glucosylation of the Biochanin A

**Fig. 7:**
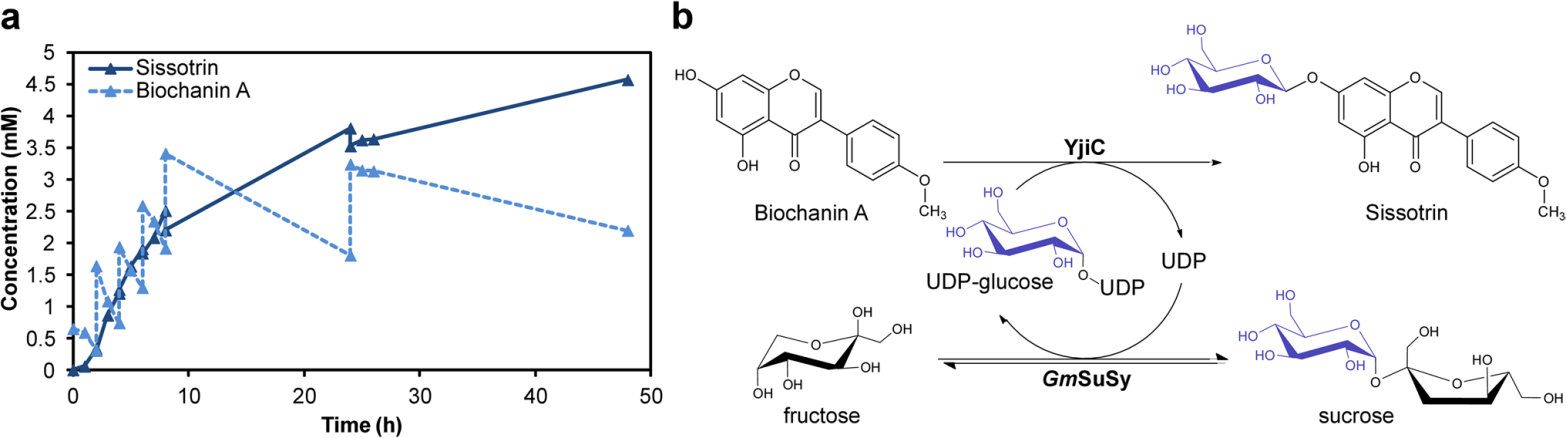
Fed-batch biocatalysts of Sissotrin by co-immobilized *Gm*SuSy and YjiC (**a**). Scheme of the glucosylation of Biochanin A by coupled YjiC-*Gm*SuSy cascade reaction (**b**)

With chosen optimal reaction conditions, enzyme activity ratio, and type of the carrier, we decided to verify our biocatalyst with isoflavone substrate (Biochanin A (Fig. 7b)), as those are characterized with very low water solubility, which might be enhanced with glucosylation. According to Han and co-workers solubility of Biochanin A reaches only 6.73 mg/L (Han et al. 2011), which prompted us to perform catalysis by implementing a fed-batch approach, to lower the precipitation of the substrate during the process. Nevertheless, each added portion of the substrate visibly escalated the opacity of the reaction mixture. During the first 8 h of the reaction, the STY kept within a range of 0.6–0.8 g/L∙h∙g of the resin, but further addition and precipitation of the poorly soluble isoflavone led to the deceleration of the reaction rate. The final STY of the Sissotrin (Biochanin A 7-*O*-glucoside) measured was 0.26 g/L∙h∙g of the resin and the total turnover number (TTN) for UDP-glucose reached up to 8.2. The structure of the obtained product was determined by NMR analysis (see Supplementary materials). The NMR spectroscopic data for the obtained product were in agreement with previously reported data (Sordon et al. 2017) (Fig. 7).

Due to proton release from nucleoside diphosphate catalyzed by glycosyltransferases, the pH of the reaction was measured just in case, to check if it could contribute to the retardation of the reaction rate. Yet, no significant pH shift was detected. After the 48 h of catalysis, the reaction mixture was separated from the resin by centrifugation, and the solid biocatalyst was washed several times on the filter, but precipitated Biochanin A prevented thorough cleaning of the resin. Hence, the downstream of the preparative catalysis of poorly soluble substrates using solid catalyst needs to be refined. The titre of the Sissotrin cumulatively reached up to 4.7 mM (1.8 g/L) (Fig. 7a) with the final conversion of 67.5% (Fig. S3, NMR analysis – Fig. S4-S8).

## Discussion

The glucosylation cascade consisting of Leloir glucosyltransferase and sucrose synthase is extensively investigated in the aspect of efficient and affordable production of the glycosides of natural products (Gutmann and Nidetzky 2016; Chen et al. 2019; Zhang et al. 2020). Current studies are focused mainly on the enhancement of robustness, increasing reaction yields, and application of a biocatalyst in an industrial environment. The co-immobilization of the enzymes is one of the employed strategies (Chu et al. 2021; Liu et al. 2021). Apart from the assets of single immobilization, such as stabilization, reusability, and facilitated downstream processing (Sheldon 2007), co-immobilization offers additional benefits. Placing several enzymes on the same carrier has a spacial proximity effect on the catalysts, which promotes the flux of the reactants through the catalytic pathway, and reduces the lag time of the reaction (Arana-Peña et al. 2021). Yet, the design of the co-immobilized biocatalysts, like all the other cascades, requires careful investigation of the optimal enzyme activity ratio, to maximize the overall biocatalyst efficiency (Siedentop et al. 2021). To omit the need for separate expression of required enzymes in two host strains followed by combining their cell lysates to attain optimal enzyme proportion or expression in a single strain with double vector expression (requiring dual selection pressure and plasmid compatibility), an expression vector with two independently induced transcriptional units was designed and employed for investigated cascade production (Fig. 1c).

On the principle, the created double expression system should enable the independent co-expression of two proteins and manipulation of their final ratio in the host cell. Yet, the screening test of the co-expression of YjiC and *Gm*SuSy from pRhaBAD-GmSuSy_Trc-YjiC plasmid with different inducer concentrations provided some unforeseen results. The cell lysates induced with higher IPTG amounts displayed higher GT and cascade activity, due to stronger induction of the YjiC expression (Fig. 2a, b). At the same time, a slightly weaker expression of the *Gm*SuSy was expected, as a result of the presumable competition between two promoters for transcriptional resources (Goodrich and McClure 1991), the toxicity of the IPTG on *E. coli* cells, which would hinder protein yield (Dvorak et al. 2015) or potential inhibition by IPTG of the RhaS as an AraC protein family member (Lee et al. 2007). Yet, a slightly higher sucrose synthase activity was detected (Fig. 2c). The continuous fluorescence measurements of the *E. coli* cultures co-expressing mCherry and GFP from analogous pRhaBAD-mCherry_Trc-GFP plasmid displayed the same pattern. The detailed analysis of the whole plasmid sequence revealed that a sequence similar to the LacO1 operator is located downstream to the RhaS’s promoter (*J23103*). Therefore, constitutively expressed LacI repressor may non-specifically bind to that region and as a result inhibits the expression of the RhaS activator, hence diminishes GmSuSy/mCherry expression. The IPTG addition depletes LacI inhibition and enhances overexpression from not only *trc* but possibly also *J23103* promoter and consequently from *rhaBAD* promoter as well. The ability of near-specific and non-specific binding of the LacI was described in the work of Garza de Leon and co-workers (Garza de Leon et al. 2017). On the other hand, the negative impact of the L-rhamnose on the overexpression from the *trc* promoter could be the result of the co-directional organization of all genes in the designed expression cassette. The LacI repressor protein sequence is located directly downstream of CDS placed under the *rhaBAD* promoter, and separated by a single T7 terminator (Fig. 1c). During the transcription from the *rhaBAD* promoter, the RNA polymerase could transcribe the following genes as well (He et al. 2020). The additional overexpression of the LacI may escalate the inhibition of the *trc* promoter (Semsey et al. 2013), although this is not the case, since a basal expression and a strong increase in fluorescence even at low IPTG concentrations were observed. Albeit, the additional non-specific binding site for LacI may to some extent reduce its pool and weaken its repression abilities. The proposed approach for tuneable co-expression of two proteins requires further improvement and investigation, e.g., *E. coli* BL21(DE3) strain is able to utilize L-rhamnose which may influence the expression from the presented expression system. Yet, it already enables co-expression of both desired enzymes in sufficient amounts and with partial control of their ratio directly at the stage of enzyme production.

The results gathered through the investigation of the optimal reaction conditions for tested enzymes agree with the literature data. The best operational activity of the *Gm*SuSy under acidic conditions comes from the pH dependence of the sucrose synthase transglycosylation between sucrose and UDP to fructose and UDP-glucose. pH between 7.5 and 9.5 prefers sucrose synthesis, whereas pH values between 5.5 and 7.5 promote UDP-glucose formation (Schmölzer et al. 2016). Yet, some reports declared the best plant SuSy activity in terms of sucrose cleavage in a neutral, not acidic environment (Baroja-Fernández et al. 2012; Liu et al. 2021). Although sucrose glycosidic bond is energy-rich, its conversion to UDP-glucose is thermodynamically disfavored (Morell and Copeland 1985). Stepwise investigation of the kinetics of sucrose cleavage by sucrose synthases revealed that only complete protonation of the UDP at pH 5.0 could shift the reaction equilibrium to UDP-glucose synthesis, which is in agreement with our observations (Gutmann and Nidetzky 2016). Known for the substrate promiscuity, YjiC glucosyltransferase (Pandey et al. 2014; Bashyal et al. 2020) was employed in several studies, where various pH values were tested. Interestingly, the product distribution of glycosylation conducted by YjiC slightly varies in different pH (Dai et al. 2018; Thapa et al. 2019), which was not observed within this research, due to the structure of substrates used, with single hydroxyl moieties available for the glucosylation. However, in most cases, a pH of 7.5 was employed as the best environment for the YjiC activity (Parajuli et al. 2014; Kim et al. 2018). Similarly, SuSy’s activity of other GTs is pH sensitive as well and depends on the difference in pK_a_ between the phosphate moiety of nucleoside diphosphate and glycosyl acceptor group (Nidetzky et al. 2018). Low pH values significantly disfavor the transfer of the glucose moiety to the acceptor molecule. The optimum operating temperature and thermostability attained for both *Gm*SuSy and YjiC correspond with other research investigations, as well as the influence of the co-immobilization on the characteristics and stability of the enzymes (Zhang et al. 2019; Chu et al. 2021; Liu et al. 2021). Temperature above 40 °C displayed denaturing impact on the enzymes in both free and immobilized form (a increased turbidity of the reaction mixture with soluble enzymes was observed after the catalysis (data not shown)). Lower activity at lower temperatures arises from lower reaction rate as prolonged incubation of the reaction mixture yielded complete conversion of the substrate *p*NP (data not shown). The vulnerability of the glycosyltransferase to divalent metal ions results from the presence of the DXD metal-binding motif, in the nucleotide-binding domain (Ünligil and Rini 2000). DXD motif coordinates divalent cations to facilitate departure of the NDP leaving group (Lairson et al. 2008). Sucrose synthase, as a member of the glycosyltransferase family (EC 2.4.1.13), was expected to respond to different Mg^2+^ concentrations, but the activity variation caused was not a limiting factor of the cascade. On the other hand, YjiC activity varied depending on the metal ion’s addition. Results for crude and immobilized YjiC clearly indicate the influence of Mg^2+^ concentration on activity; however, without a deeper analysis, we cannot provide a clear explanation of the mechanisms behind. We can only speculate that for YjiC, Mg^2+^ concentration may play a role in protein structural changes, as for the immobilized enzyme the effect of concentration is not evident. Thapa and co-workers reported that different metal ions influence the product distribution of the YjiC glucosylation (Thapa et al. 2019), similarly to the pH effect on the GT regioselectivity mentioned earlier. Additionally, lesser effect of Mg^2+^ concentration on the cascade may be related to the instability of the nucleotide sugars in the presence of Mg^2+^ at alkaline pH (Baroja-Fernández et al. 2012). It would explain lesser shifts in the cascade activity at different Mg^2+^ concentrations compared to single enzyme glucosylation—recycling of the UDP-glucose guarantees continuous synthesis of the sugar donor and autonomize the process from UDP-glucose stability. Analysis of the optimal reaction conditions was performed using strain harboring pRhaBAD-GmSuSy_YjiC plasmid for the enzyme production; however, cell lysates obtained from double controlled expression plasmid should share similar cascade optimal conditions, despite the difference in enzyme ratio.

The co-immobilization of His_6x_-tagged YjiC glucosyltransferase and *Gm*SuSy sucrose synthase was performed on the basis of the immobilized metal affinity chromatography (IMAC) technique. The Ni^2+^ are the most widely used ions for this purpose since they display both fine affinity and specificity. Yet, nickel toxicity (Zhang et al. 2015) hinders its use in the production of pharmaceutically relevant compounds. The EziG resins coated with non-toxic Fe^3+^ to date were not employed for the immobilization of the glycosyltransferase class of enzymes, and their characterization at a given application is beneficial. The analysis of the monitored immobilization parameters showed that in comparison to Ni-agarose resin, all three EziG carriers exhibited high immobilization yields (~100%) (Table 3). All tested resins exhibited acceptable recovered activity (~50%) (Table 3). The depletion in the observable activity of the carriers versus initial cell lysate activity could be caused by the substrate diffusion limitation (i.e., the substrates were quicker utilized by the enzymes than they diffuse into the carrier pores) (Bolivar et al. 2013) or the wrong oriented co-immobilization. Rocha-Martin and co-workers reported that the co-localization of the co-immobilized enzymes is a crucial factor for the enzyme activity preservation on the carrier particles (Trobo-Maseda et al. 2020). Their investigation showed that depending on the immobilization strategy, expressed activity of the immobilized biocatalyst varied between 45 and 97% of the initial enzyme solution activity. For the satisfying cascade co-localization, Nidetzky and co-workers applied Z_basic2_ protein as a protein tag, which guaranteed homogenous distribution of enzymes in polymeric ReliSorb SP400 carrier, and fine recovered activity (up to 91%). They also demonstrated good recyclability of prepared solid biocatalyst —60% of remaining initial activity after 15 screening cycles (each 15 min long) or 40% of remaining initial activity after 15 preparative cycles (each 12 h long) (Liu et al. 2021). Nevertheless, non-ordered co-immobilization of the YjiC-*Gm*SuSy cascade on the CPG EziG Opal carrier displayed also very good reusability (up to 12 cycles), without any significant loss of activity (< 10%) (Fig. 6), which compensates for average recovered activity extending the cascade activity lifetime. At this juncture, it has to be pointed out that *p*NP is not the most suitable substrate for the YjiC glucosyltransferase, and it has been chosen only for the biocatalyst characterization, due to easy detection of the glucosylation progress by colorimetric measurements. Yet, satisfying STY of *p-*nitrophenol (5.7 g/L∙h∙g of Opal resin) was achieved, which proved the co-immobilized cascade effectiveness.

The verification of the efficiency and applicability of the designed biocatalyst to larger-scale processes by the Biochanin A glucosylation also brought insights into the further required optimization steps. The usage of solid catalysts generally facilitates the purification and enables the reuse of the enzymes, but in the case of poorly soluble reactants, the resin recovery stage can be a challenging task. Precipitated substrate aggregated on the carrier particles, disabled full separation, and probably disrupted the flux of the reactants between enzymes, what might affect the final reaction yield. The application of the co-solvents may simplify the process, as well as the reaction rate, by enhancing the availability of the substrate to the catalyst. Nonetheless, the final 67.5% conversion of 68.75 mg of Biochanin A resulted in the production of ~73 mg of the Sissotrin (0.26 g/L∙h∙g of resin), which happen to be the highest yield for Biochanin A 7-*O*-glucoside achieved by the enzymatic reaction so far.

To sum up, the glycosylation cascade made from glycosyltransferase and sucrose synthase has unlocked the potential for a highly precise and versatile enzymatic glycosylation of small molecules (Elling 1997; Bungaruang et al. 2013). Promiscuous YjiC glucosyltransferase and highly active *Gm*SuSy sucrose synthase were successfully co-expressed and co-immobilized directly from the cell lysate on CPG-based EziG carriers, bringing an efficient and reusable biocatalyst that enables glucosylation of poorly soluble isoflavone. The designed double-controlled expression system consisting of two independently induced expression cassettes brought new insights into the possible relationships between the transcriptional machinery involved in the expression of recombinant proteins and may be used as a guide for the further design of the dual-expression plasmid architecture.

## Supplementary information

Below is the link to the electronic supplementary material.

Supplementary file1 (PDF 1180 KB)

## Data Availability

The authors confirm that the data supporting the findings of this study are available within the article (and/or) its supplementary materials.
