# Supplementary material for: Evaluation of double expression system for co-expression and co-immobilization of flavonoid glucosylation cascade
Source: Appl Microbiol Biotechnol. 2022 Nov 5;106(23):7763–78. doi: 10.1007/s00253-022-12259-5 (PMC9668961; doi:10.1007/s00253-022-12259-5)
Supplement: Supplementary file 1 — Supplementary file1 (PDF 1180 KB) [file 253_2022_12259_MOESM1_ESM.pdf]

Applied Microbiology and Biotechnology

Supplementary material

Evaluation of double expression system for co-expression and co-immobilisation of flavonoid glucosylation cascade

Agata Matera[1], Kinga Dulak[1], Sandra Sordon[1], Kacper Waśniewski[1], Ewa Huszcza[1], Jarosław Popłoński[1*]

[1]Department of Food Chemistry and Biocatalysis, Wrocław University of Environmental and Life Sciences, C.K. Norwida 25, 50-375 Wrocław, Poland

***Corresponding author:**
jaroslaw.poplonski@upwr.edu.pl, phone: +48 71 320 5197

Table S1. Genes, primers, plasmids and *E. coli* strains used within this study

Genes, primers, plasmids and strains	Properties	Source or reference
**Gene**		
GmSuSy	sucrose synthase from *Glycine max*	Genbank: AAC39323.1, OP381218
yjiC	glucosyltransferase from *Bacillus licheniformis*	Genbank: AE017333.1, OP381219
mCherry	mCherry	Shener et al. 2004
GFP	*GFPmut3** mutant of WT with the following mutations: S2R,S65G,S72A	Cormack et al. 1996
**Primers**		
PS1	AGGGCGGCGGATTTGTCC	Silva-Rocha et al., 2013
PS2	GCGGCAACCGAGCGTTC	Silva-Rocha et al., 2013
YjicFwd	GGTCTCAAATGGGCCATAAACATATCGCCATC	This study
YjicRev	GGTCTCTCGAATTTAACGCCTGCCGGTGCC	This study
SuSyGm_middle_Fwd	GAATCCGGATGCACTGCAG	This study
SuSyGm_middle_Fwd2	CAAGCCTGCTGGCACATAAAC	This study
SuSyGm_middle_Fwd3	CGACCTTTGCAACCTGTAATGG	This study
Plasmids		
pRhaBAD_cassette	pUC ori, AmpR, *rhaS*, *rhaBAD* promoter, T7 terminator	This study
pTrc_cassette	pUC ori, AmpR, *lacI*, *trc* promoter, T7 terminator	This study
pSEVA23g19g1	pBBR1 ori, KmR	Data not publish
pSEVA23g19g2	pBBR1 ori, KmR	Data not publish
pRhaBAD_12	pSEVA23g19g1 carring pRhaBAD_cassette	This study
pTrc_23	pSEVA23g19g2 carring pTrc_cassette	This study
pSEVA63g19gA	pBBR1 ori, GmR	Data not publish
pSEVA182-T7RBS_BC	pUC ori, AmpR, T7 RBS	Data not publish
pSEVA182- N-His$_{6x}$-tag _CD	pUC ori, AmpR, N-His$_{6x}$-tag	Data not publish
pMA-T-GmSuSy_DG	ColE1 ori, AmpR, *GmSuSy*	This study
pMA-T-YjiC_DG	ColE1 ori, AmpR, *YjiC*	This study
pSEVA182-mCherry_DG	pUC ori, AmpR, *mCherry*	Data not publish
pSEVA182-GFP_DG	pUC ori, AmpR, *GFP*	Data not publish
pRhaBAD-GmSuSy	pRhaBAD_12 carrying N-His$_{6x}$-*GmSuSy*	This study
pTrc-YjiC	pLacI-trc_23 carrying N-His$_{6x}$-*YjiC*	This study
pRhaBAD-GmSuSy_Trc-YjiC	pSEVA63g19gA carrying N-His$_{6x}$-*GmSuSy* under *rhaBAD* promoter and N-His$_{6x}$-*YjiC* under *trc* promoter	This study
pRhaBAD-mCherry	pRhaBAD_12 carrying T7 RBS, N-His$_{6x}$-*mCherry*	This study

pTrc-GFP	pLacI-trc_23 carrying T7 RBS, N-His$_{6x}$-*GFP*	This study
pRhaBAD-mCherry_Trc-GFP	pSEVA63g19gA carrying N-His$_{6x}$-*mCherry* under *rhaBAD* promoter and N-His$_{6x}$-*GFP* under *trc* promoter	This study
pRhaBAD-GmSuSy_YjiC	pSEVA63g19gA carrying N-His$_{6x}$-*GmSuSy* and N-His$_{6x}$-*YjiC* under *rhaBAD* promoters, *rhaS*	This study
**Strains**		
DH5-alpha	F′ proA$^+$B$^+$ lacI^q Δ(lacZ)M15 zzf::Tn10 (Tet^R) / fhuA2Δ(argF-lacZ)U169 phoA glnV44 Φ80Δ(lacZ)M15 gyrA96 recA1 relA1 endA1 thi-1 hsdR17	New England Biolabs Inc.
BL21 (DE3)	fhuA2 [lon] ompT gal (λ DE3) [dcm] ΔhsdS<br>λ DE3 = λ sBamHIo ΔEcoRI-B int::(lacI::PlacUV5::T7 gene1) i21 Δnin5	New England Biolabs Inc.
10-beta	Δ(ara-leu) 7697 araD139 fhuA ΔlacX74 galK16 galE15 e14- ϕ80dlacZΔM15 recA1 relA1 endA1 nupG rpsL (Str^R) rph spoT1 Δ(mrr-hsdRMS-mcrBC)	New England Biolabs Inc.

Construction of pRhaBAD-GmSuSy_YjiC plasmid

Coding sequences of *GmSuSy* and *yjiC* were cut out from pMA-T-GmSuSy_DG and pMA-T-YjiC_DG plasmids by *Bsa*I-HFv2 restriction enzyme. *GmSuSy* coding sequence was inserted along with T7 RBS and N-His$_{6x}$-tag to pRhaBAD_12 plasmid. *YjiC* coding sequence was inserted along with *rhaBAD* promoter, T7 RBS, N-His$_{6x}$-tag and T7 terminator to pSEVA23g19g2. Next, both plasmids were cut by *Bbs*I restriction enzyme and expression cassettes were inserted alongside into pSEVA63g19gA to create pRhaBAD-GmSuSy_YjiC plasmid, with each genes under the control of *rhaBAD* promoter.

Table S2. The composition of 2xM9 minimal medium. The listed quantities are required for preparation of 1 L of the medium

**Chemical**	**Amount**
$Na_2HPO_4 \cdot 12H_2O$	34.2 g
KH_2PO_4	6 g
NaCl	1 g
NH_4Cl	2 g
$MgSO_4 \cdot 7H_2O$	0.5 g
D-glucose	8 g
$CaCl_2 \cdot 2H_2O$	0.015 g
$Fe_2(SO_4)_3 \cdot xH_2O$	0.074 g
$EDTANa_2 \cdot 2H_2O$	0.072 g
Trace metals solution[a]	0.1 ml

[a]Trace metals solution contained: 8.5 g $ZnCl_2$, 18 g $CuSO_4 \cdot 5H_2O$, 14 g $MnCl_2 \cdot 4H_2O$, 18 g $CoCl_2 \cdot 6H_2O$ in 1 L of H_2O.

Preparation of the resins

Ni-agarose resin A 50% *(v/v)* solution in ethanol of nickel resin was transferred into 25 mL Eppendorf falcon and settled by centrifugation at 500 g for 5 min. The supernatant was removed and resin was equilibrated twice by 5 volumes of binding buffer (50 mM HEPES, 50 mM KCl, 300 mM NaCl, 20 mM imidazole, pH 7.5), followed by centrifugation at 500 g for 5 min and removal of the supernatant.

EziG resins Resins were weighed into 25 mL Eppendorf falcons, suspend in binding buffer (50 mM HEPES, 50 mM KCl, 300 mM NaCl, 20 mM imidazole, pH 7.5), and stirred at 100 rpm agitation on the orbital shaker (ELMI, Riga, Latvia) for 5 min. Next, resins were settled down by centrifugation at 1000 g for 2 min, and supernatants were removed.

BCA assay solution

The colour reagent was prepared by combining 49 parts of the bicinchoninic acid alkaline solution (Sigma-Aldrich, St Louis, USA) and 1 part of copper(II) sulfate 4% *(w/v)* solution (Sigma-Aldrich, St Louis, USA) with 5 mM of glutamic acid.

BCA assay evaluation

The effect of the sucrose on the BCA assay for the reducing sugar detection was assessed by preliminary measurements of different fructose concentrations (0, 0.05, 0.1, 0.2, and 0.5 mM in reaction buffer (50 mM HEPES, 50 mM KCl, pH 7.5) and reaction buffer supplemented with 500 mM of sucrose. The results indicate slight effect on the absorbance (therefore possible cross-interaction), although no significant impact on measurement deviation.

Table S3 Fructose concentrations measured in the reaction buffer and reaction buffer with 500 mM of sucrose. The standard deviations were obtained from three individual replicates

Fructose concentration	0	0.05	0.1	0.2	0.5
Fructose concentration measured in the reaction buffer	0.029±0.004	0.062±0.004	0.106±0.004	0.192±0.014	0.459±0.035
Fructose concentration measured in the reaction buffer with 500 mM sucrose	0.029±0.001	0.067±0.003	0.112±0.010	0.216±0.010	0.520±0.037

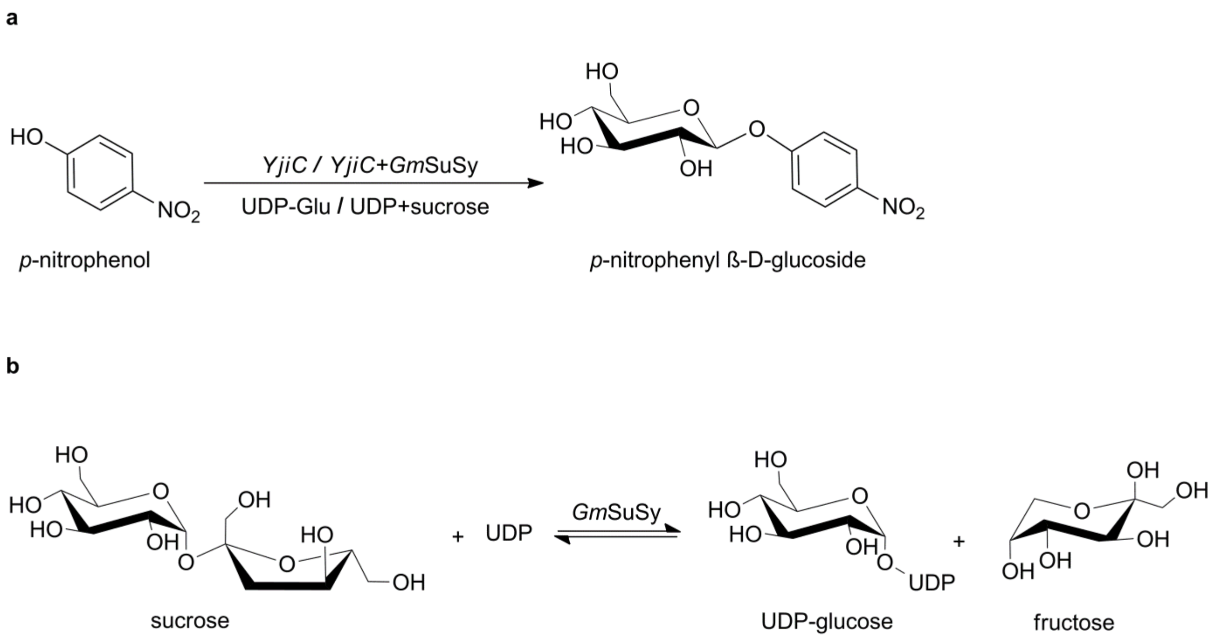

Figure S1. Schemes of test reactions employed for glucosyltransferase and cascade (**a**) and sucrose synthase (**b**) characterization. Abbreviations: UDP - uridine diphosphate; UDP-Glu - uridine diphosphate glucose

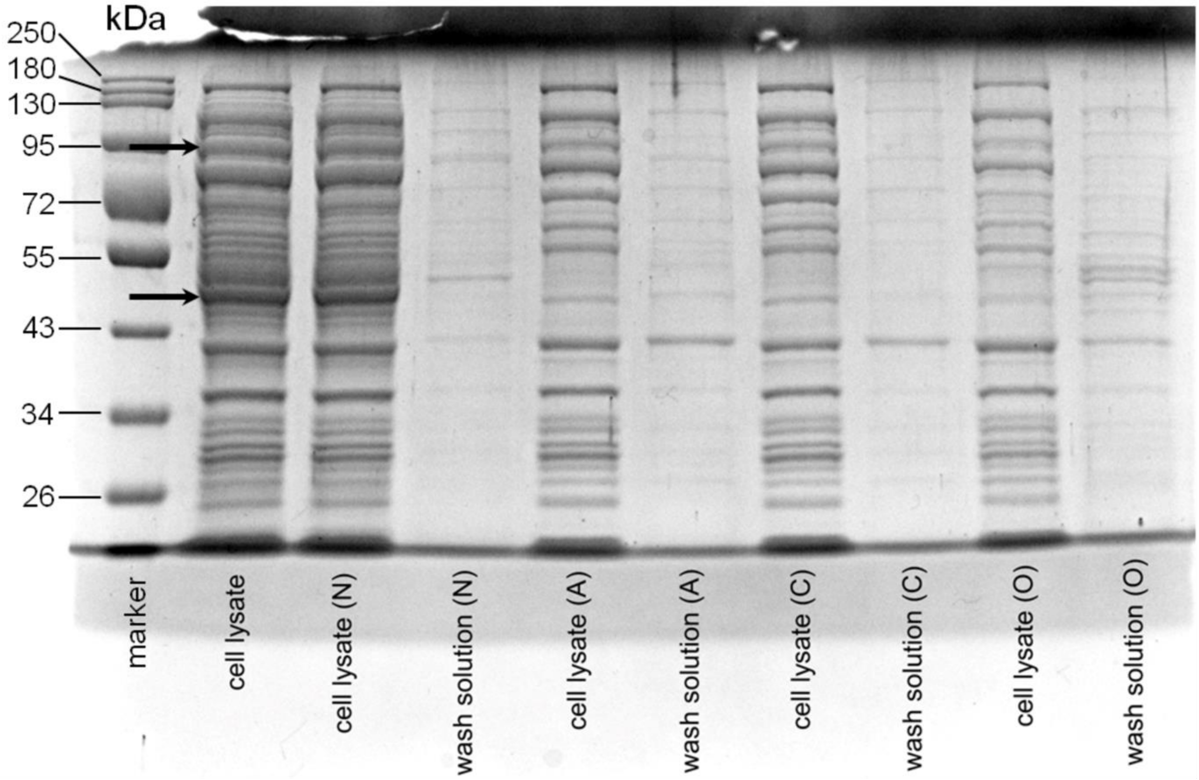

Figure S2. SDS-PAGE gel presenting proteins contained in cell lysates before and after the immobilization procedure, and in wash solutions. The upper arrow correspond to *Gm*SuSy band (92 kDa) and the lower to YjiC (45 kDa), Abbreviations: N-Ni-agarose resin; A – Amber resin; C – Coral resin; O – Opal resin

Purification of the product

The reaction product of Biochanin A conversion was extracted from reaction mixture (40 mL) using 3x30mL of

ethyl acetate. Organic fraction was evaporated to dryness and the product was separated from remaining substrate using column chromatography on silica gel 60 (~40 g, 230–400 mesh, Merck, Darmstadt, Germany) using cyclohexane: 2-propanol (9:1 -> 1:1 *v/v*) mixture gradient as an eluent.

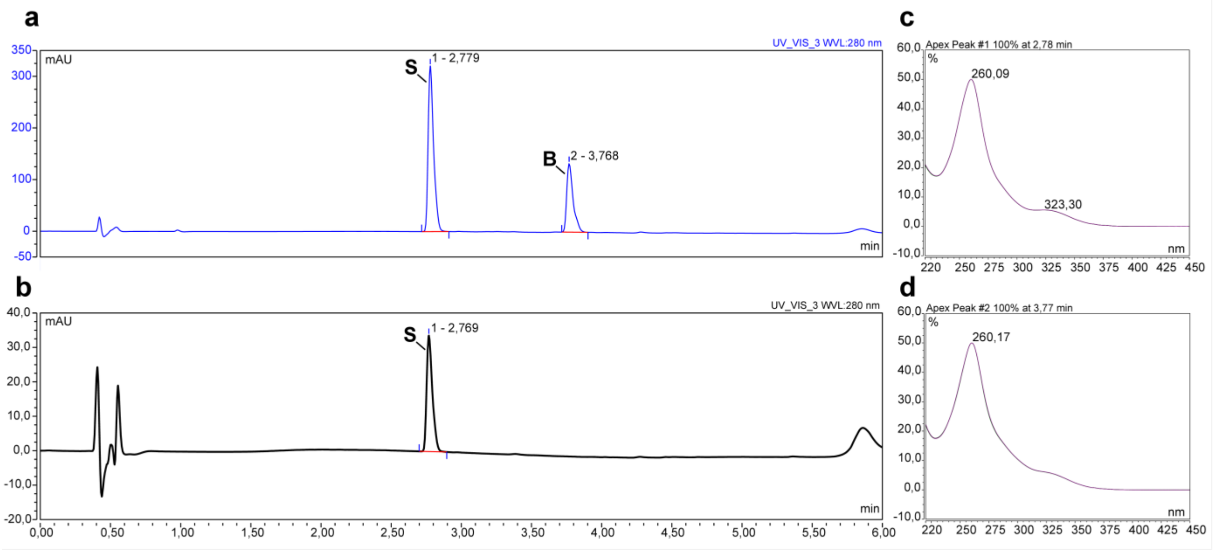

Figure S3. UHPLC analysis of the mixture of semi-preparative glucosylation of Biochanin A after 48 h of the catalysis (**a**), and purified Sissotrin (**b**). UV-Vis spectra of Biochanin A (**c**) and Sissotrin (**d**). Abbreviations: B – Biochanin A; S – Sissotrin

Identification of the product

Purified product was identified by NMR analysis in DMSO-d_6: ^{1}H-NMR, ^{13}C-NMR, ^{1}H-^{1}H-NMR (COSY) and ^{1}H-^{13}C-NMR (HSQC, HMBC), which were recorded on a DRX Bruker Avance TM 600 (600 MHz) instrument.

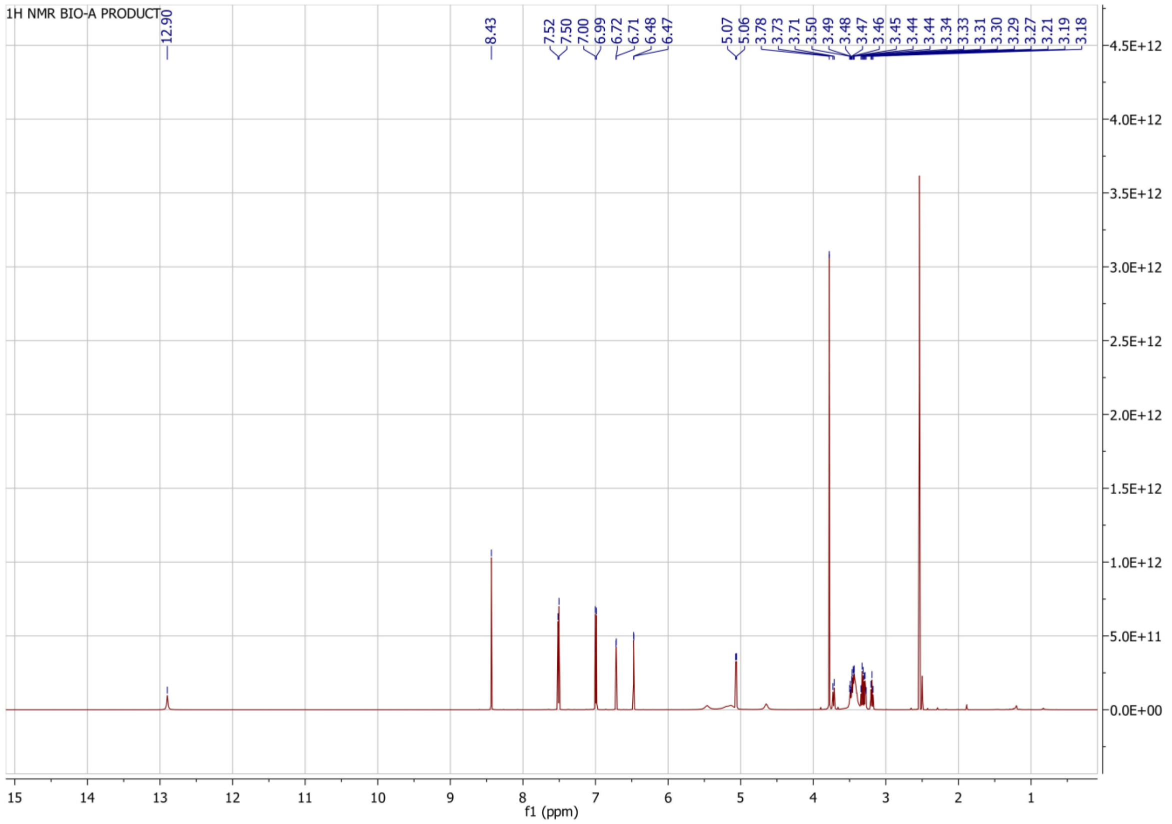

Figure S4. ^{1}H-NMR spectra of Sissotrin obtained by semi preparative glucosylation of Biochanin A

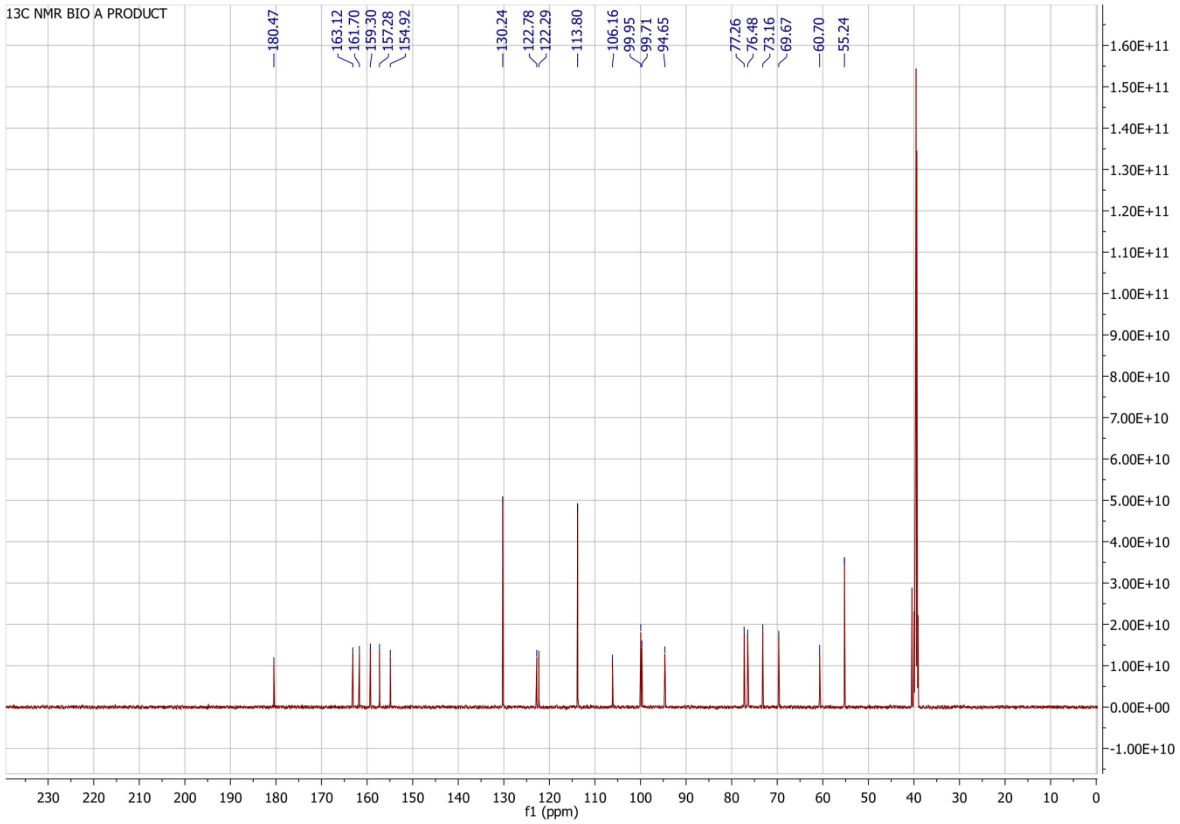

Figure S5. ^{13}C-NMR spectra of Sissotrin obtained by semi preparative glucosylation of Biochanin A

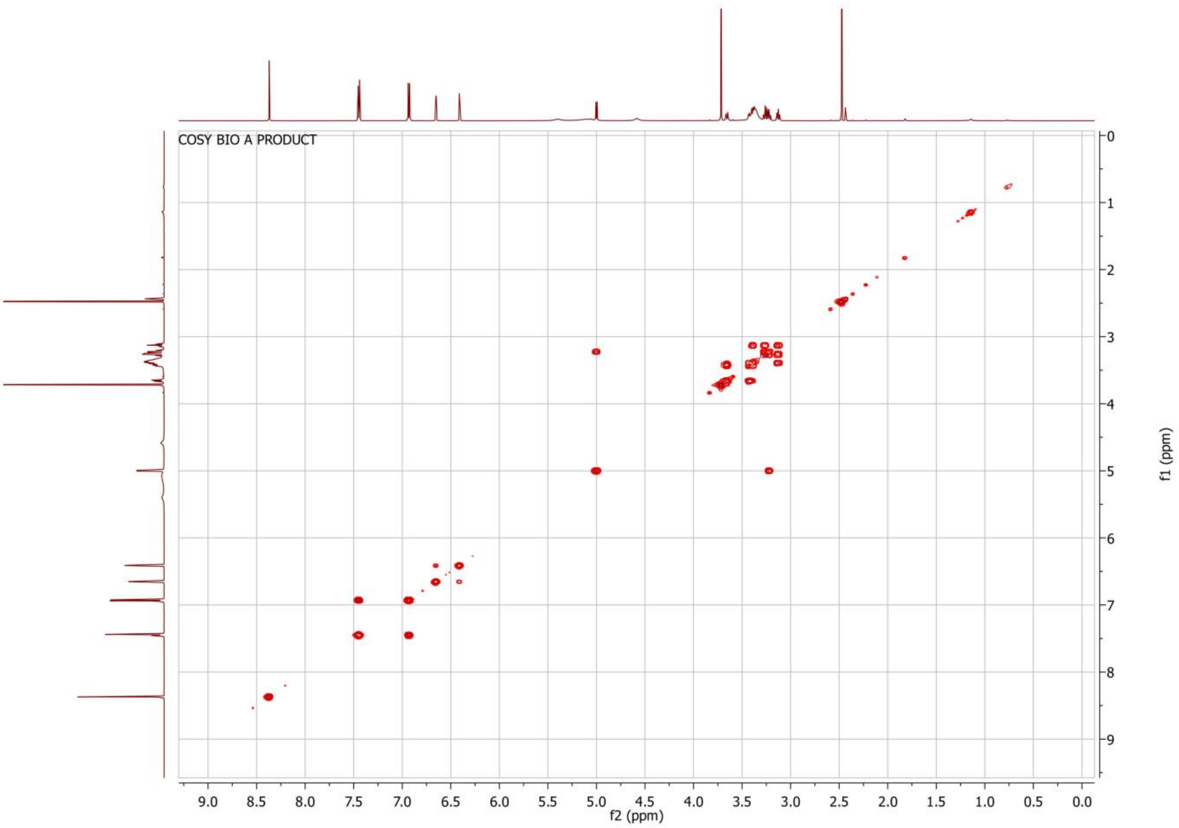

Figure S6. COSY spectra of Sissotrin obtained by semi preparative glucosylation of Biochanin A

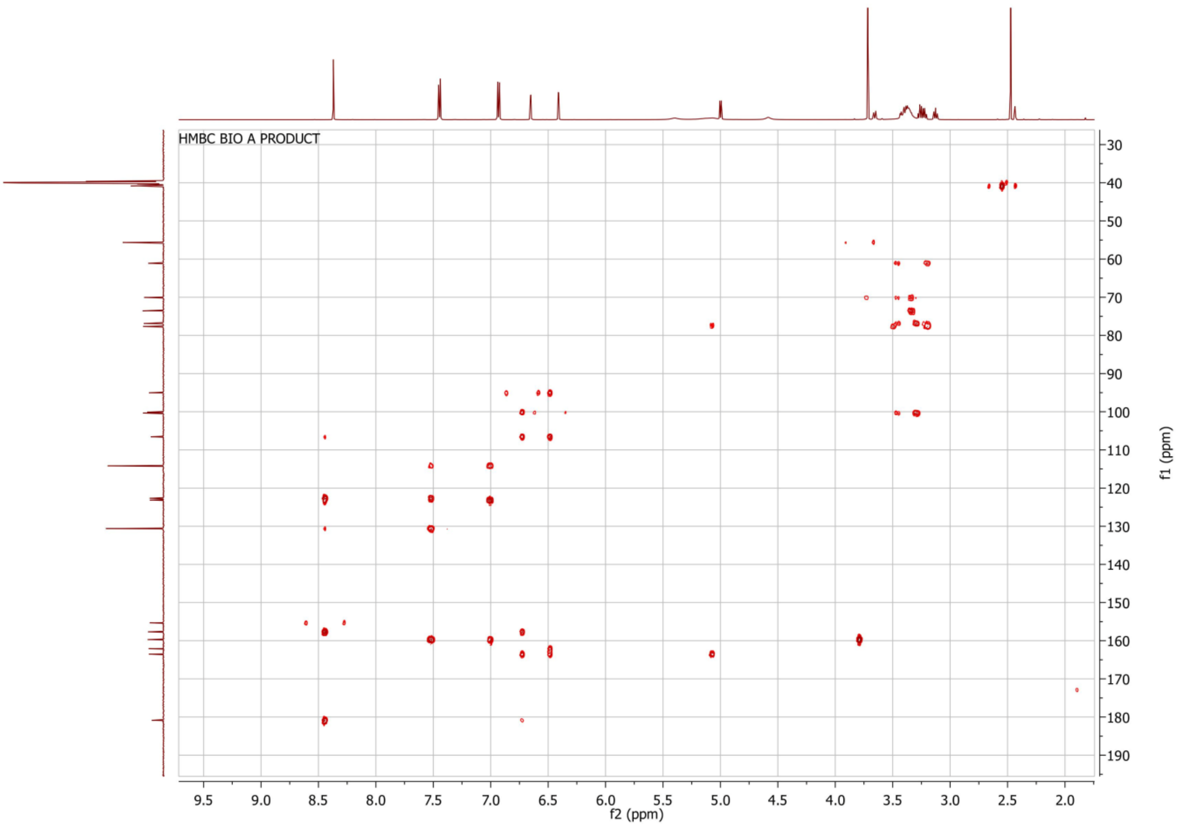

Figure S7. HMBC spectra of Sissotrin obtained by semi preparative glucosylation of Biochanin A

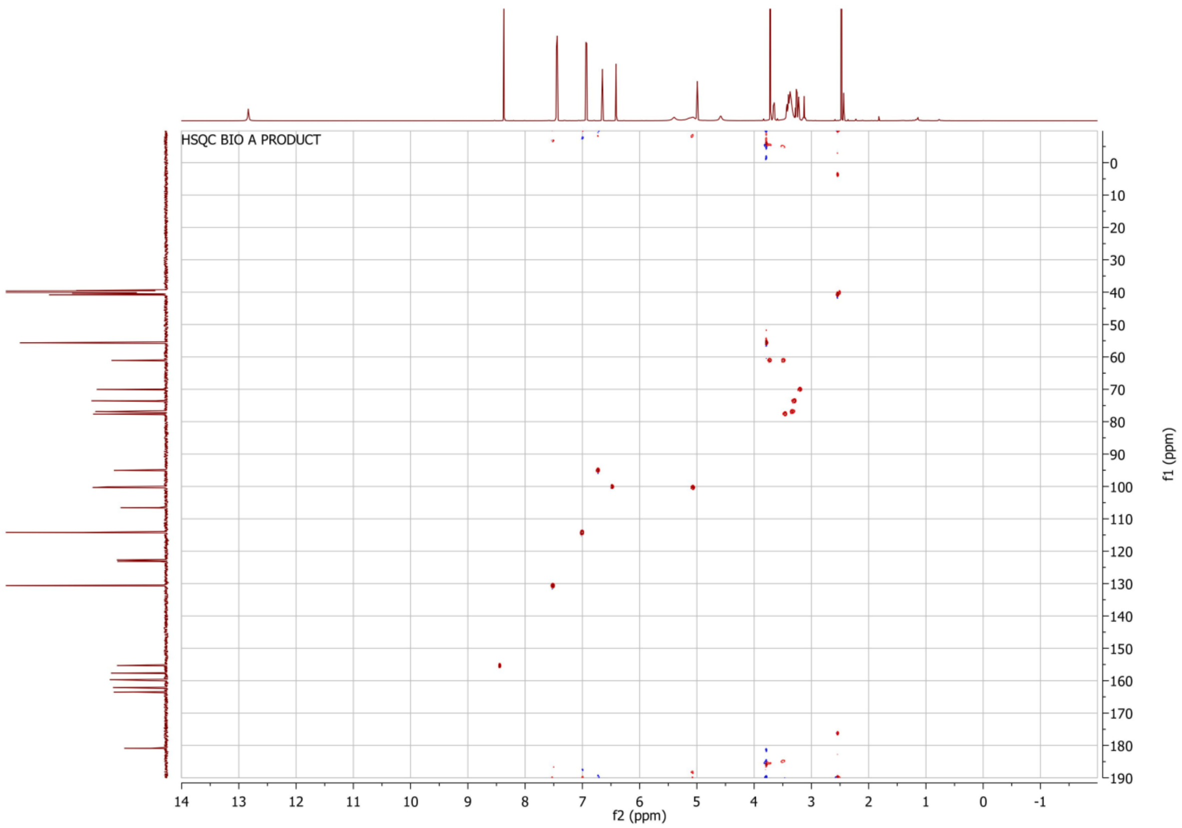

Figure S8. HSQC spectra of Sissotrin obtained by semi preparative glucosylation of Biochanin A

References

Silva-Rocha, R., Martínez-García, E., Calles, B., Chavarría, M., Arce-Rodríguez, A., De Las Heras, A., Páez-Espino, A. D., Durante-Rodríguez, G., Kim, J., Nikel, P. I., Platero, R., & De Lorenzo, V. (2013). The Standard European Vector Architecture (SEVA): a coherent platform for the analysis and deployment of complex prokaryotic phenotypes. *Nucleic Acids Research*, *41*(D1), D666–D675. https://doi.org/10.1093/NAR/GKS1119
